# No evidence of early life resource pulse effects on age‐specific variation in survival, reproduction and body mass of female Siberian flying squirrels

**DOI:** 10.1111/1365-2656.14218

**Published:** 2024-11-11

**Authors:** C. Le Coeur, V. Berger, V. Lummaa, R. Wistbacka, V. Selonen

**Affiliations:** ^1^ Department of Biology University of Turku Turku Finland; ^2^ Centre for Ecological and Evolutionary Synthesis (CEES), Department of Biosciences University of Oslo Oslo Norway; ^3^ Department of Biology University of Oulu Oulu Finland

**Keywords:** ageing, disposable soma theory, fast‐living species, masting, resource allocation, senescence, silver‐spoon hypothesis

## Abstract

1. Understanding the diversity and causes of senescence patterns in the wild remains a challenging task, in particular among fast‐living species for which senescence patterns have been poorly studied. Early life environmental conditions can shape senescence by influencing trade‐offs between early and late life performance (disposable soma theory) or individual fitness through lifelong positive effects (silver spoon effects).
2. Using a 23‐year‐long monitoring dataset of two populations of Siberian flying squirrels (*Pteromys volans L.*) in western Finland, we analysed the occurrence, onset and rate of senescence in female survival, body mass and reproductive performance. We also examined how early life pulsed resources (tree masting during the year of birth) influence age‐specific variations in these traits.
3. Our results indicate that survival senescence occurs after sexual maturity from 3 years of age. Females experiencing high resource availability at birth tended to survive better, but the age‐specific trend was not affected by early life resource conditions. Maternal body mass declined slightly with age, starting at 4 years, regardless of early resource conditions. Similarly, among reproductive traits, we showed late‐onset senescence in both litter size and annual reproductive probability, with no evidence supporting an effect of early life resources on these trends. We found no decline in juvenile body mass or in the juvenile size‐number trade‐off with maternal age.
4. Our findings suggest that pulsed resources experienced at birth have a limited long‐lasting impact on the life‐history traits of this fast‐living rodent, with no significant effect on senescence patterns.

## Graphical abstract

Multiple theories have emerged to explain how early life conditions can influence senescence rates, including the disposable soma theory and the silver spoon hypothesis. However, few studies have explored these theories in natural resource‐pulse environments, in particular among fast‐living mammals for which senescence patterns have been poorly documented. This study provides new evidence for survival, body mass and reproductive senescence in two wild populations of a fast‐living rodent, the Siberian flying squirrel. However, no effects of early life resource conditions, measured by the highly variable production of catkins from alder and birch masting, were detected on senescence patterns. Photo by Henrik Lund.

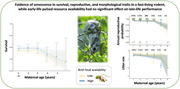

## 1 INTRODUCTION

Senescence, defined as the decline in functional abilities with age (Monaghan et al., 2008), affects most species in nature (Jones et al., 2008; Nussey et al., 2013; Reinke et al., 2022). However, not all organisms senesce at the same rate and/or in the same way. The patterns of senescence vary substantially across and within species (Jones et al., 2014; Reinke et al., 2022), and understanding the causes of such diversity remains a major challenge in the wild. Both the pace of life and allometric variation across organisms have been identified as important predictors of differences in the onset and rate of senescence (Jones et al., 2008; Lemaître et al., 2020; Reinke et al., 2022; Vágási et al., 2021). Classical evolutionary ageing theories (Medawar, 1952; Williams, 1957) predict faster and earlier onset of senescence in populations of small and/or fast‐living species. These species experience higher risk of extrinsic mortality and a higher reproductive investment early in life than species located at the slow end of the slow–fast continuum of life histories. However, most empirical studies on senescence in the wild have been conducted on mammalian and avian species (Reinke et al., 2022; Zajitschek et al., 2020 for tetrapod ectotherms and wild insects) that occupy the slow end of the slow‐fast continuum (Lemaître et al., 2020; Shefferson et al., 2017). Survival, reproductive or body mass senescence among wild populations of fast‐living species have been much less studied, and strong empirical evidence of senescence is currently lacking.

Among‐ and within population variation in age‐specific patterns are affected in part by the variation in local environmental conditions experienced at some stages or throughout the life of the individuals (Cooper & Kruuk, 2018; Nettle et al., 2013; Sanghvi et al., 2022). For instance, early food availability (Hammers et al., 2013; Mumby et al., 2015), early population density (Nussey et al., 2007), early adult number of helpers (Berger et al., 2018), early life predation risks (Balbontín & Møller, 2015), and adult life pack size (Stahler et al., 2013) have been found to influence late‐life performance and senescence patterns. However, most of these previous studies consider only one or a few fitness‐related traits, which only partially reflect the integrative and complex process of senescence. Measuring several fitness‐related traits (i.e. survival, reproductive, morphological and physiological traits; e.g. Descamps et al., 2008; Hayward et al., 2015; Walker & Herndon, 2010) is essential to characterise the causes and consequences of senescence in the wild.

The disposable soma theory posits that early life investment in reproduction or growth, at the cost of maintenance and repair, accelerates senescence and reduces lifespan due to accumulated cellular damage (Kirkwood, 1977; Kirkwood & Rose, 1991; Lemaître et al., 2015). The degree to which higher early life investment in reproduction is favoured over somatic maintenance, survival, or future reproduction, is expected to vary according to environmental conditions, such as natal environmental conditions. This theory predicts that both unfavourable and favourable early life conditions could shift the optimal trade‐off between early and late‐life investments, potentially accelerating senescence. For instance, female red deer (*Cervus elaphus*) born in years of high population density exhibited higher reproductive potential early in life and faster reproductive senescence than those born in years of high food availability (Nussey et al., 2007). Conversely, female collared flycatchers (*Ficedula albicollis*) raised in low‐competition environments (favourable conditions) invested more in early life reproduction than those raised in high‐competition environments, at the cost of faster senescence (Spagopoulou et al., 2020). The impact of unfavourable conditions on reproductive timing and senescence can also be explained by a different mechanism. According to the internal predictive adaptive response theory (iPAR; Nettle et al., 2013), individuals facing adverse early life conditions may adjust their reproductive timing, favouring earlier reproduction to maximise fitness in anticipation of reduced late‐life performance. Alternatively, the silver spoon hypothesis (Grafen, 1988) predicts that individuals experiencing favourable environments early in life might benefit from relatively good conditions when reaching adulthood, leading to lifelong positive effects on a multitude of aspects of individual fitness, including late life performance (e.g. Cooper & Kruuk, 2018).

Although several evolutionary hypotheses have been identified, testing them in natural settings remains challenging due to confounding factors. For example, individuals with access to greater and higher‐quality food early in life often occupy better territories, which may lead to more favourable conditions later in life (e.g. reduced mortality risk). Systems characterised by pulsed resources—where the production of abundant resources is intermittent—offer a unique opportunity to investigate the effects of early life resource conditions on age‐specific patterns under relatively controlled conditions. Some mammal species, such as arboreal rodents, are nutritionally dependent on these pulsed resources (Boutin et al., 2006; Selonen & Wistbacka, 2016; Wauters et al., 2008). Individuals born in high or low food availability experience vastly contrasting resource conditions in their life, which influence reproduction and survival (Allain et al., 2024; Hämäläinen et al., 2017; Hoset et al., 2017; Le Coeur et al., 2018), and are likely to impact senescence. To our knowledge, few studies have examined the effects of early pulsed resources on senescence in rodents or mammals in general (Allain et al., 2024; Descamps et al., 2008; Petrullo et al., 2024).

**FIGURE 1:**
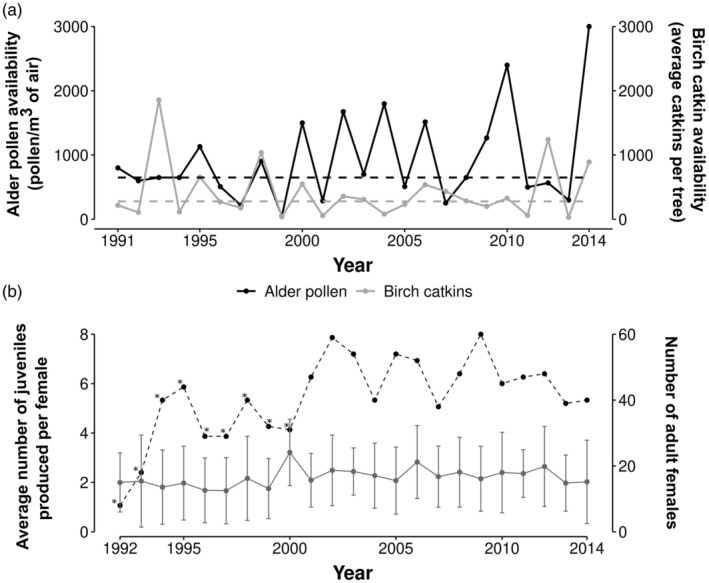
(a) Annual variation in two landscape‐level food availability estimates: Alder pollen (accumulated sums of average daily counts of airborne pollen/m^3^ during spring; black line) and birch catkins (average catkins per tree counted in the previous winter; grey line) over 24 years, 1991–2014. We used indices for western Finland that were sampled in Vaasa. Black and grey dashed lines refer to the median value of alder pollen availability and birch catkin availability, respectively. (b) Annual variation in the number of juveniles produced per female (mean ± standard deviation; dark grey points) and the total number of adult females monitored at Vaasa and Luoto study sites (dashed line). Asterisks indicate years of lower monitoring effort in Luoto.

Using 23 years of longitudinal data from two nest‐box breeding populations of Siberian flying squirrels (*Pteromys volans L*.)—a fast‐living mammal and pulsed resource consumer—this study aims to examine the patterns of senescence in female survival, body mass, three reproductive traits and the offspring size‐number trade‐off. We also test the disposable soma theory (or iPAR) and the silver‐spoon hypothesis by assessing whether early environmental conditions, particularly the pulsed resources experienced during the birth year, influence age‐specific variation in these traits. In these populations, Siberian flying squirrels depend on catkin production from deciduous trees (alder and birch), which form a pulsed food resource for the species (Figure 1; Hoset et al., 2017; Jokinen et al., 2019; Selonen & Mäkeläinen, 2017). Based on life history theories of ageing, we predicted a fast and early onset of senescence for some or all fitness‐related traits considered of this fast‐living species, starting at sexual maturity. Under the silver spoon hypothesis, we expected that favourable food availability at birth would have positive effects throughout life, including a slower rate of maternal survival, body mass and/or reproductive senescence. According to the disposable soma theory, if high or low food availability during early life resulted in higher rates of early life reproduction or investment in body mass, we expected a faster rate of senescence in late life. Although the trade‐off between offspring size and number is well‐documented (Smith & Fretwell, 1974; Stearns, 1992), less is known about the influence of maternal age and early life resources on this trade‐off. Maternal age (Pettifor et al., 1988, as maternal state; McNamara & Houston, 1996) and resource acquisition (MacNulty et al., 2009; Rutz et al., 2006; van Noordwijk & de Jong, 1986) are both expected to influence the optimal number of offspring produced and their mass. We thus hypothesized that the strength of the size‐number trade‐off would increase with maternal age and/or when mothers experienced lower resources at birth.

## 2 MATERIALS AND METHODS

### 2.1 Study species

The Siberian flying squirrel is a fast‐living, arboreal rodent inhabiting spruce‐dominated boreal forests from Finland to eastern Siberia. Female flying squirrels are territorial, mostly living in nonoverlapping home ranges (on average 7 ha), while males live in larger and overlapping home ranges (on average 60 ha; Selonen et al., 2013). They can breed after their first year of life, producing 1–2 litters per year, in April and June, with on average 2–3 offspring per litter (Figure 1). After the first litter born in April, approximately 15% of females produce summer litters in the study areas (Selonen & Wistbacka, 2016), especially when spring food availability is high (Selonen et al., 2016). Breeding dispersal in adult females is practically absent (Selonen & Wistbacka, 2017), and individuals as old as 7 years have been recorded in the study populations. Mean juvenile and adult survival were estimated to be around 0.15–0.20 and 0.35–0.50, respectively (Brommer et al., 2017). Main predators of flying squirrels are larger owls, such as the Ural owl (*Strix uralensis*), the Eurasian eagle owl (*Bubo bubo*), the goshawk (*Accipiter gentilis*) and the European pine marten (*Martes martes*; Selonen & Mäkeläinen, 2017).

### 2.2 Study population and monitoring

Flying squirrels were monitored by capture–mark–recapture methods using nest boxes at two study sites along the coast of West Finland from 1992/1993 to 2014, Luoto (63°49′ N, 22°49′ E) and Vaasa (63°3′ N, 22°41′ E). Luoto study site is a peninsula (44 km^2^) dominated by shoreline spruce (*Picea abies*) dominated mixed forests, clear‐cuts and cultivated Scots pine (*Pinus sylvestris*) forests. Vaasa study site is dominated by spruce forest patches, clear‐cuts and agricultural fields and covers an area of 4 km^2^, which was extended to 25 km^2^ after 2000. More information on the study areas is provided by Selonen et al., 2014.

During the study period, approximately 250 nest boxes were placed in sets of 2–4 within forest patches of various sizes, as defined by satellite images (*c*. two boxes per hectare). Occupancy rate by flying squirrels was low (*c*. 25%), indicating that available boxes were not a limiting factor in the populations. Boxes were checked in June each year when both females and juveniles were in the nest. They were checked again in August, depending on the presence of breeding females in June but also infrequently throughout the year. Flying squirrels use boxes for roosting and may use more than one box to raise broods. The number of second litters might be underestimated if females changed nest box after the first brood. However, within female territories, multiple nest boxes were present, and we are unaware of any observations of females giving birth in dreys, as litters have only been observed in nest boxes or rare natural cavities (own observations).

Female adults and juveniles were captured by hand in nest boxes, sexed, weighed and marked with ear‐tags (Hauptner 73850, Hauptner, Germany). Females flying squirrels sharing a nest box with juveniles were presumed to be their mothers. The fate of juveniles after leaving the nest was not known for most individuals. This study did not require ethical approval, but the permit to ear‐tag flying squirrels was provided by Finnish Centre for Environment (ELY Centre: permit numbers: Dnr LSU‐2002‐L‐265(254), Dnr LSU‐2007‐L‐573(254), DnrEPOELY/412/07.01/2012).

### 2.3 Food availability data

Flying squirrels feed on deciduous trees that occur within or at the edge of spruce forests. At our study sites, birch (Betula spp.), alder (Alnus spp.) and aspen (*Populus tremula*) are the main forage items (Selonen & Mäkeläinen, 2017; Selonen & Wistbacka, 2016). During winter and early spring, birch and alder catkins are their main food sources, and birch catkins constitute the main part of the winter diet. However, alder catkins are preferred over birch (Selonen & Mäkeläinen, 2017). Catkin production varies considerably between years and forms a highly pulsed resource in the forest (Figure 1). Pulsed resource strongly influences the reproduction of flying squirrels at our study sites, by increasing the likelihood of second litters and the lifetime reproductive success of females (Hoset et al., 2017; Selonen & Wistbacka, 2016).

To investigate the influence of food availability at birth on age‐specific patterns, we used both alder and birch availability in spring each year (Figure 1). Birch catkin production was sampled annually in winter in Vaasa by the Finnish Forest Research Institute and was used as a proxy for both study sites (Hokkanen, 2000). As a proxy of alder catkin production, we used aerial pollen data sampled in Vaasa by the aerobiology unit at the University of Turku (Ranta et al., 2008; Selonen et al., 2016). The data consisted of accumulated sums of average daily counts of airborne pollen/m^3^ of air during spring.

After scaling the annual estimates of masting alder and birch resource availability separately (Pearson's *r* = 0.23; Figure 1), we averaged the two scaled variables per year. Low values refer to years with low alder and birch catkin production available in spring, while high values refer to mast years for both resources. We used this composite variable during the first year of life (referred to as ‘birth food availability’) to test for early life effects on age‐specific patterns. Food availability from year to year is strongly negatively correlated (lag‐1 serial correlation coefficient = −0.53; Figure 1 and Figure S1 for annual variation of the composite variable).

### 2.4 Dependent variables and data selection

We investigated the senescence patterns and the influence of birth food availability on age‐specific variation in maternal survival, body mass, three reproductive traits, including the *annual reproductive probability, litter size* and *juvenile body mass*, and the *offspring size‐number trade‐off*. Traits were estimated from 1 year of age. Out of 570 female flying squirrels, 189 and 381 were marked as juveniles and adults, respectively, between 1992 and 2014. Adult females were considered yearlings when first captured due to their high site fidelity and low breeding dispersal (Hanski et al., 2000; Selonen & Wistbacka, 2017), as observed in other sciurids (e.g. Gaudreau‐Rousseau et al., 2023; Wauters & Dhondt, 1993). Age at marking was accounted for in the statistical analyses (see details in the next section). *Maternal body mass* (*n*_body masses_ = 1097; *n*_females_ = 491) was estimated based on 1–5 annual records (median = 1), of which more than 89% were taken during the reproductive season. We quantified the *annual reproductive probability* (0 if a female did not reproduce in either breeding season and 1 if it reproduced in one or both seasons; *n*_reproductive events_ = 782, *n*_mothers_ = 489). We were not able to account for variation within the reproductive season in maternal body mass. We analysed *litter size*, defined as the number of juveniles per litter (from 0 to 4+ juveniles; *n*_litters_ = 779, *n*_mothers_ = 489) and *juvenile body mass*, measured individually before weaning. As female flying squirrels reproduced primarily during the first reproductive season of the year, we only considered the first litter of the year to analyse age‐specific variation in juvenile body mass (*n*_records_ = 1506, from *n*_mothers_ = 421). In a second step, we tested whether the *trade‐off between body mass and number of juveniles in a litter* changed depending on maternal age.

### 2.5 Statistical analyses

Annual survival (*phi*) and recapture (*p*) probabilities were estimated with Cormack‐Jolly‐Seber models (CJS) in RMark (Laake, 2013). Prior to model selection, we performed a goodness of fit analysis in R2ucare (Gimenez et al., 2018) to test the assumptions of equal survival and equal catchability of marked females at each study site. No assumption violations or overdispersion were detected (variance inflation factor c‐hat <1; details in Supporting information). We explored age‐specific survival probabilities using age as a continuous variable by constraining the survival trajectory at each age as Gompertz or logit‐linear functions. Starting from the general model *p*(time)*phi*(time), we considered a sequential step‐by‐step approach by testing age and group effects (including birth food availability, monitoring effort, age at marking and site) on *p*, then used the most parsimonious models of *p* to model the survival probability (*phi*; Table 1 and Tables S8 and S9). Model selection was based on Akaike's information criterion corrected for small sample size (AICc). A threshold modelling was applied to estimate the onset of senescence, using five scenarios with breakpoints ranging from age 1 to age 5 (estimating two slopes: *β*_age.pre‐onset_ and *β*_age.post‐onset_ before and after the age of onset). We compared these threshold models to two candidate models, including age as a linear or quadratic covariate, and ranked them by their AICc values. Using the best model from the previous step, we assessed the effect of birth food availability on survival senescence, controlling for food availability at year *t* and other covariates, including age at marking (two levels: juvenile or adult) and site.

**TABLE 1:** Linear, quadratic and threshold models testing the effect of age on survival, maternal and juvenile body mass, annual reproductive probability (ARP), litter size and juvenile mass‐number trade‐off.

Trait	Model	Other covariates	df	AIC_c_	ΔAIC_c_	*ω*	Dev.
Survival	**Threshold_Age3**	AnnualFood + site + MarkingAge	**6**	**1380.34**	**0.00**	**0.39**	**477.45**
	**Threshold_Age2**		**6**	**1381.20**	**0.86**	**0.25**	**478.31**
	Quadratic		7	1382.68	2.35	0.12	477.77
	Linear		6	1383.20	2.86	0.09	480.31
	Threshold_Age4		6	1383.50	3.17	0.08	480.62
	Constant		5	1385.22	4.89	0.03	484.36
	Threshold_Age5		6	1385.38	5.04	0.03	482.49
Maternal body mass	**Quadratic**	AnnualFood + AFR + ALR + site + MarkingAge + ReproSeason	**14**	**9404.41**	**0.00**	**0.47**	**9376.02**
	**Threashold_Age4**		**14**	**9405.23**	**0.82**	**0.31**	**9376.84**
	Threashold_Age2		14	9406.41	2.00	0.17	9378.02
	Threashold_Age3		14	9409.62	5.21	0.03	9381.23
	Threashold_Age5		14	9411.25	6.84	0.02	9382.86
	Linear		13	9414.63	10.22	0.00	9388.29
	Constant		12	9447.70	43.29	0.00	9423.42
ARP	**Threshold_Age5**	AnnualFood + AFR + ALR + site + MarkingAge	**10**	**313.24**	**0.00**	**0.29**	**287.97**
	**Threshold_Age4**		**10**	**313.36**	**0.12**	**0.27**	**289.54**
	**Linear**		**9**	**314.31**	**1.07**	**0.17**	**295.50**
	Threshold_Age2		10	315.39	2.15	0.10	241.97
	Quadratic Age		10	316.07	2.83	0.07	294.50
	Threshold_Age3		10	316.22	2.98	0.06	295.94
	Constant		8	316.95	3.71	0.04	194.46
Litter size	**Threshold_Age5**	AnnualFood + AFR + ALR + site + MarkingAge	**13**	**2169.11**	**0.00**	**0.59**	—
	Threshold_Age3		13	2172.45	3.34	0.11	—
	Linear		12	2173.27	4.16	0.07	—
	Threshold_Age4		13	2173.30	4.19	0.07	—
	Quadratic Age		13	2173.55	4.44	0.06	—
	Constant		11	2173.64	4.53	0.06	—
	Threshold_Age2		13	2175.11	6.00	0.03	—
Juvenile body mass	**Quadratic Age**	AnnualFood + AFR + ALR + site + MarkingAge + LitterSize + DaySinceJan1	**14**	**10176.64**	**0.00**	**0.96**	**10148.36**
	Threshold_Age4		14	10183.80	7.16	0.03	10155.52
	Threshold_Age3		14	10185.98	9.34	0.01	10157.70
	Threshold_Age2		14	10189.43	12.79	0.00	10161.15
	Constant		12	10208.69	32.05	0.00	10184.48
	Linear		13	10209.34	32.70	0.00	10183.10
Trade‐off	Age* N_**juv**_ **+ N**_**juv**_^**2**^	AnnualFood + AFR + ALR + site + MarkingAge + LitterSize + DaySinceJan1	**14**	**10213.52**	**0.00**	**0.46**	**10185.24**
	**N**_**juv**_ **+ N**_**juv**_^**2**^ * Age		**14**	**10214.4**	**0.88**	**0.30**	**10186.12**
	Age* (N_**juv**_ **+ N**_**juv**_^**2**^ **)**		**15**	**10215.26**	**1.74**	**0.19**	**10184.94**

*Note*: Linear mixed models were fitted for maternal and juvenile body mass, with mother ID, year of observation and forest patch included as random factors. Mother's birth year was also treated as a random factor in the analysis of maternal body mass. Generalised linear mixed models (binomial distribution) were used to model ARP, with female ID and year as random effects. For litter size, zero‐inflated generalised linear models (Conway–Maxwell Poisson distribution) were fitted. AIC_c_ estimates corrected for small sample size, ΔAICc, model weight (ω) and deviance (except for litter size) are presented. The top models (ΔAICc < 2) are shown in bold.

Abbreviations: AFR, age of first reproduction; ALR, age of last reproduction; AnnualFood, food availability in year *t*; DaySinceJan1, day of juvenile body mass measurement during the reproductive season; ReproSeason, “1” if the female was weighed during the reproductive period and “0” otherwise; MarkingAge, marked as juveniles or as adults (yearlings); N_juv_, litter size; Threshold_AgeX, threshold model with a breakpoint at age X; time, years of monitoring.

As a general approach to investigating senescence and the effects of early environmental conditions in each body mass and reproductive trait, we ran a set of candidate models that included age as a linear, quadratic or threshold term (from age 2 to age 5), and compared the AICc between models (Burnham & Anderson, 2004). AFR and ALR, the age at which a female was first and last known to reproduce, were included as covariates in the models to assess the relative contribution of selective appearance and disappearance, respectively (van de Pol & Wright, 2009). Various covariates, specific to each trait and described below, were incorporated into the models. Based on the best AICc model, we tested for the disposable soma theory and silver‐spoon hypothesis by examining the additive effect and two‐way interaction between birth food availability and age, while controlling for adult food availability in year *t*.

To analyse senescence in maternal and juvenile body mass and in the trade‐off between juvenile body mass and number, we fitted linear mixed models with mother ID, forest patch (to control for spatial heterogeneity) and year of observation as crossed random factors. For maternal body mass, the mother's birth year was also treated as a random factor to account for cohort effects. We added a 2‐level factor (reproductive vs. non‐reproductive seasons) as fixed effect to account for weight gain during pregnancy. For the analysis of juvenile body mass and body mass‐number trade‐off, litter size and the day of measurement for each juvenile (‘DaySinceJan1’) were included as fixed factors. Day of measurement was used to account for intra‐annual variation in the date of birth and date of body mass measurement among juveniles (details in Supporting information). Zero‐inflated generalised linear mixed models with a Conway–Maxwell Poisson distribution were used to investigate the age‐specific variation in litter size. For the analysis of annual reproductive probability (ARP), we used generalised linear mixed models with a binomial distribution. To avoid convergence problems, only female ID and year of observation were treated as random factors. In all models, we included study site and age at marking as fixed factors. All continuous variables were standardised to compare the magnitude of the coefficients (bootstrapped 95% CI and likelihood profile 95% CI for litter size and ARP) between age, ALR and AFR. For details and exceptions in the models, see Table 1 and Supporting information. All statistical analyses were conducted using R version 4.4.1 (R Core Team, 2024).

## 3 RESULTS

### 3.1 Senescence and early life food availability effects on age‐specific variation in survival

**FIGURE 2:**
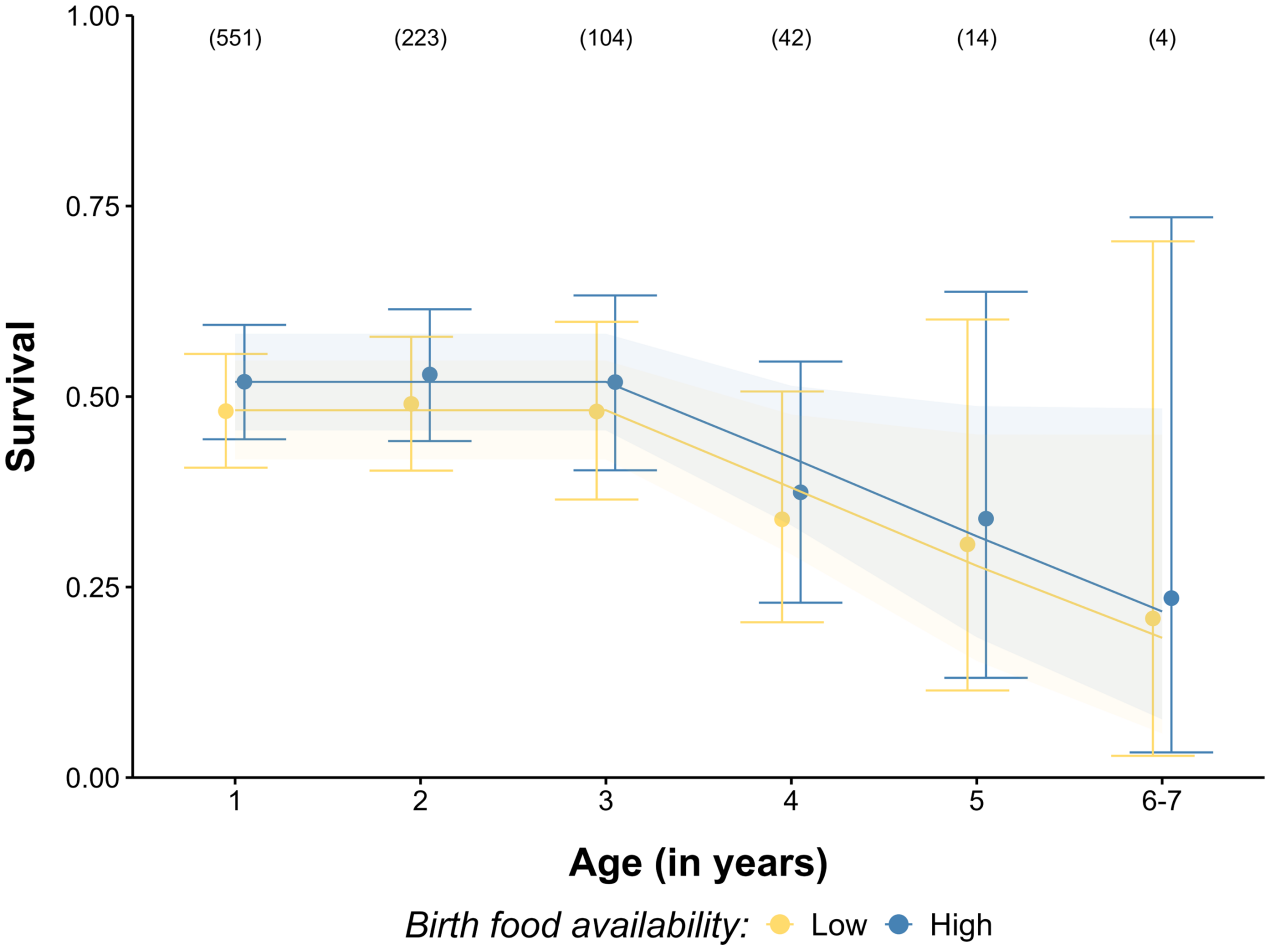
Age‐dependent estimates of annual female survival probabilities (circles; 95% confidence intervals are indicated by vertical bars) according to food availability experienced at birth. For Figures 2, 3, 4, birth food availability was transformed into a discrete variable with two factors, below and above the median in yellow and blue, respectively. Lines represent the estimates from the continuous model *phi* (Threshold_Age3 + BirthFood + MarkingAge)*p*(.), assuming a Gompertz function (shaded areas indicate 95% CI). Numbers at the top indicate sample sizes.

Equal support was found for two threshold models (Table 1), revealing a decline in survival with age, starting at 2 to 3 years of age (*β*_0[intercept]_ = 0.40, 95% confidence intervals (CI): 0.21, 0.60; *β*_age.post‐onset_3years_ = −0.31, 95% CI: −0.53, −0.10 on the loglog scale, Figure 2). Deviance and AICc of Gompertz and logit‐linear models did not differ significantly. When fitting a 3‐year threshold model, we found some support for the effects of birth food availability (ΔAICc < 2, Table 2 and Table S9). Females born in a high‐food environment tend to survive better at adulthood than females born in a low‐food environment but no effect on senescence rate was detected (*β*_birthFood_ = 0.12, 95% CI: −0.03, 0.26; Figure 2). Female individuals marked as yearlings exhibited, on average, slightly lower survival rates than those marked as juveniles (*β*_MarkingAge_ = −0.19, 95% CI: −0.38, 0.004; Figure S7). Site did not influence maternal survival (*β*_[Site = Vaasa]_ = −0.07, 95% CI: −0.25, 0.12). The probability to recapture individuals in our populations was found to be constant and equal to 0.87 (95% CI: 0.82, 0.92; Table 1, model selection in Table S8).

**TABLE 2:** AIC model selection table for early life pulsed resource effects (‘BirthFood’) on age‐specific variation of maternal survival (constant recapture probability), body mass, annual reproductive probability (ARP), litter size, juvenile body mass and juvenile size‐number trade‐off.

Trait		Model	df	AIC_c_	ΔAIC_c_	Dev.
Survival	M1_a_	Age_pre‐onset_Age3_ + Age_post‐onset_Age3_ + BirthFood	7	1379.79	0.00	1365.67
	M2_a_	Age_pre‐onset_Age3_ + Age_post‐onset_Age3_	6	1380.34	0.54	477.45
	M3_a_	Age_pre‐onset_Age3_ + Age_post‐onset_Age3_ * BirthFood	8	1380.79	0.99	1364.63
Maternal body mass	M1_b_	Quadratic (Age + Age^2^)	14	9404.41	0.00	9376.02
	M2_b_	Age * BirthFood + Age^2^	16	9405.61	1.20	9373.11
	M3_b_	Age + BirthFood * Age^2^	16	9405.96	1.55	9373.46
	M4_b_	Age_pre‐onset_Age4_ + Age_post‐onset_Age4_	14	9405.23	0.00	9376.84
	M5_b_	Age_pre‐onset_Age4_ * BirthFood + Age_post‐onset_Age4_	16	9406.61	1.38	9374.10
ARP	M1_c_	Age	9	314.31	0.00	295.50
	M2_c_	Age + BirthFood	10	314.41	0.10	294.12
	M3_c_	Age * BirthFood	11	315.36	1.05	293.02
	M4_c_	Age_pre‐onset_Age4_ + Age_post‐onset_Age4_	10	313.36	0.00	289.54
	M5_c_	Age_pre‐onset_Age4_ + Age_post‐onset_Age4_ + BirthFood	11	313.50	0.14	290.02
	M6_c_	Age_pre‐onset_Age4_ * BirthFood + Age_post‐onset_Age4_	12	314.88	1.52	290.48
	M7_c_	Age_pre‐onset_Age4_ + Age_post‐onset_Age4_ * BirthFood	12	315.19	1.83	290.44
Litter size	M1_d_	Age_pre‐onset_Age5_ + Age_post‐onset_Age5_ + BirthFood	14	2165.24	0.00	—
Juvenile body mass	M1_e_	Quadratic (Age + Age^2^)	14	10176.64	0.00	10148.36
	M2_e_	Age + Age^2^ + BirthFood	15	10178.47	1.83	10148.15
Trade‐off	M1_f_	Age * N_juv_ + N_juv_^2^	14	10215.65	0.00	10187.37
	M2_f_	Age * N_juv_ + N_juv_^2^ + BirthFood	15	10217.59	1.94	10187.27

*Note*: The covariates included in the models for each trait are detailed in Table 1. Age_pre‐onset_ and Age_post‐onset_ correspond to age before and after the onset of senescence in threshold model, respectively. AIC_c_ estimates corrected for small sample size, ΔAIC_c_ and deviance are presented. Only the first models (ΔAIC_c_ < 2) are given (see full AIC_c_ model selections in Supporting information). Abbreviations indicated in Table 1.

### 3.2 Senescence and early life food availability effects on maternal body mass

**FIGURE 3:**
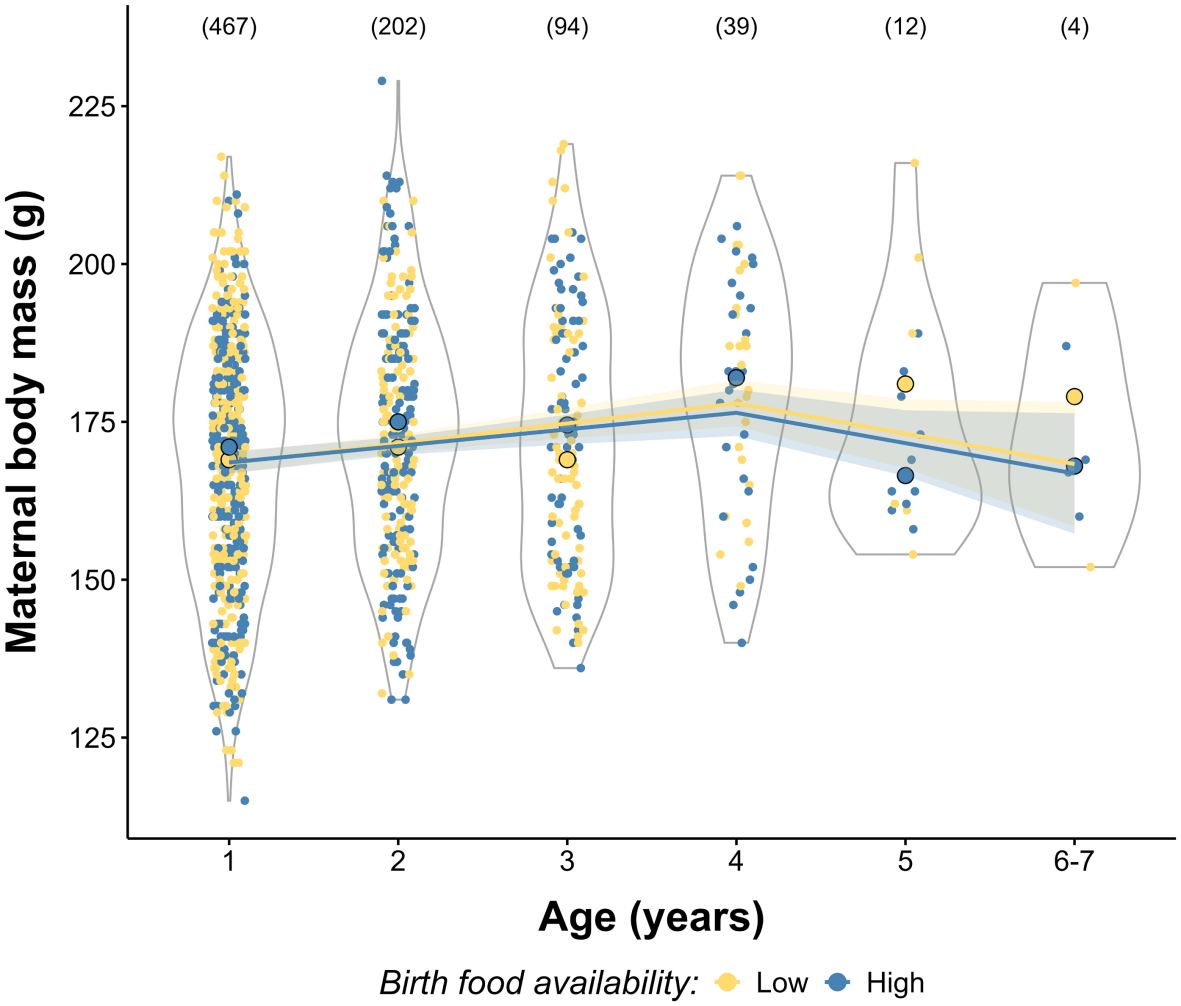
Variation in maternal body mass (in grams) according to age. Solid lines and shades, respectively, represent the model predictions and 95% CI (from model M5_b_ in Table 2; Figure S2 for predictions from the quadratic model M2_b_). Violins give the distribution of the observed values according to birth food availability (below and above the median in yellow and blue, respectively). Yellow and blue dots with black outlines represent the median maternal body mass at each age for each food availability category. The number of unique females for each age is shown at the top.

Model comparisons of age‐related variation in maternal body mass supported both quadratic and threshold models with an onset of senescence at 4 years of age (ΔAICc < 2; Table 1; full model selection in Table S1). Maternal body mass slightly increased as flying squirrels get older, peaking at the age of 3–4 years and decreased from age 4 (*β*_Age_ = 6.19, 95% CI: 4.40, 8.00; *β*_Age2_ = −1.40, 95% CI: −2.20, −0.61 from the quadratic model; *β*_age.pre‐onset_ = 4.76, 95% CI: 3.40, 6.15; *β*_age. post‐onset_ = −1.16, 95% CI: −2.32, −0.01 from the threshold model; Figure 3 and Figure S2). The interaction between maternal age and birth food availability was selected in the best‐supported models, but no statistical support was found for differences in senescence between females experiencing low or high food availability at birth (Table 2 and Table S1; Figure 3). We found a negative effect of AFR (age of first reproduction; *β*_AFR_ = −3.27, 95% CI: −4.56, −1.98) and no effect of ALR (age of last reproduction; *β*_ALR_ = −0.03, 95% CI: −1.57, 1.53). Females weighed on average [SD] 170.9 g [19.8] (*n* = 1124) and 155.3 g [12.4] (*n* = 153) during the reproductive and non‐reproductive seasons, respectively, with year‐to‐year variation, from 156.0 g [24.2] to 179.1 g [23.1] during the reproductive season. On average, females had a higher mean body mass in Luoto than in Vaasa study area (*β*_[site = Vaasa]_ = −9.18, 95% CI: −11.72, −6.61).

### 3.3 Senescence and early life food availability effects on reproductive performance

**FIGURE 4:**
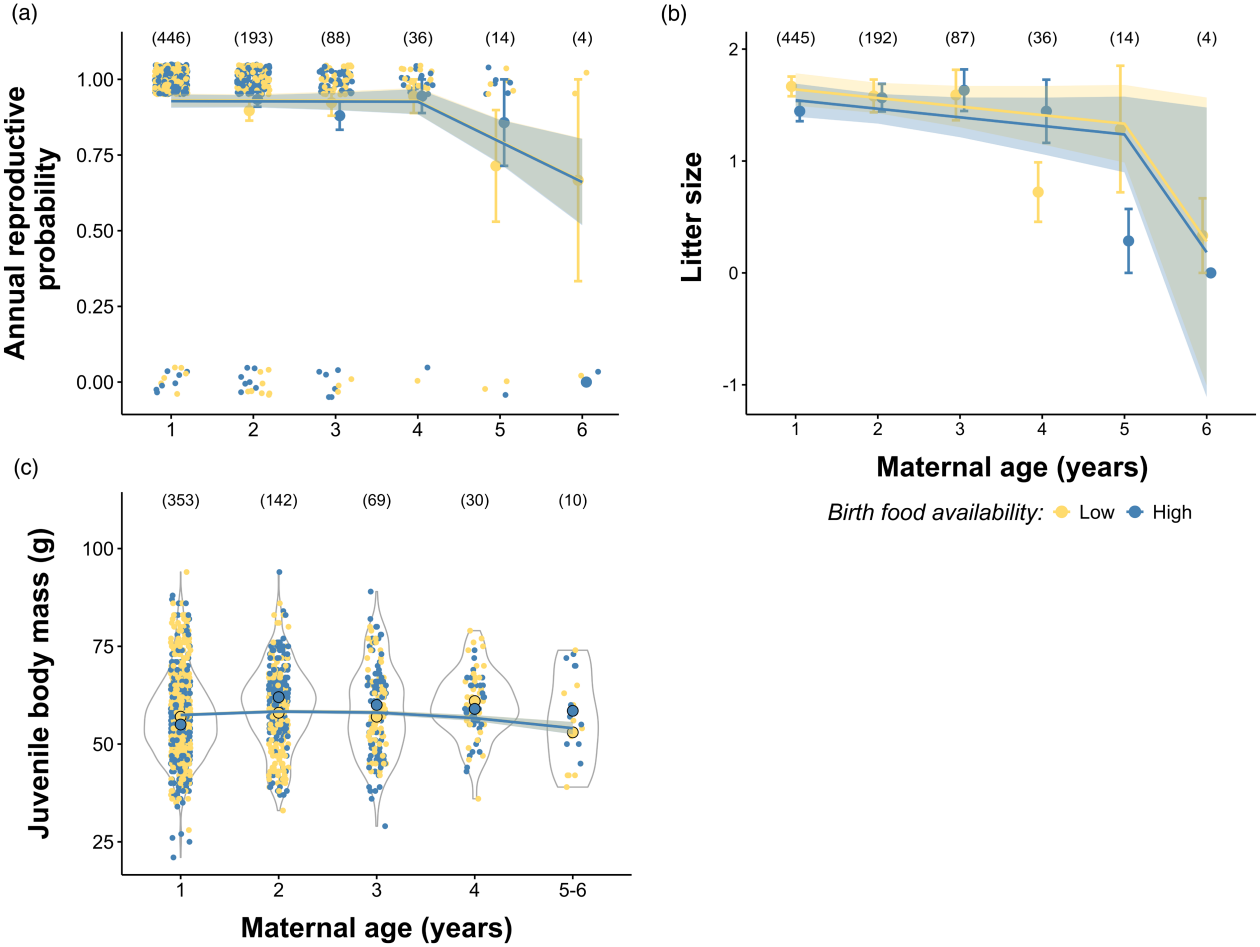
Variation in annual reproductive probability (a), litter size (b) and juvenile body mass before weaning (c) as a function of maternal age and food availability experienced by the mothers at birth (below and above the median, represented in yellow and blue, respectively). The lines and shaded areas indicate model predictions and 95% CI from (a) the threshold model M5_c_, (b) the threshold model M1_d_ and (c) the quadratic model M2_e_ in Table 2. The number of unique mothers is shown at the top of each panel. In (a) and (b), mean values are shown as circles ± standard errors. In (a) and (c), observed values at each age, according to birth food availability, are represented by yellow and blue dots. The median juvenile body mass (c) is indicated by filled dots with black outlines.

The average proportion of females that reproduced each year was high in the populations (mean [SD] = 0.94 [0.21]). Our analysis, based on the best‐selected models, revealed either an onset of senescence at age 4–5 (*β*_age.pre‐onset_ = −0.18, 95% CI: −0.72, 0.34; *β*_age. post‐onset_ = −1.57, 95% CI: −3.08, −0.29 model M4_c_ in Table 1; Figure 4a) or a continuous linear decline in annual reproductive probability with age (*β*_0_ = 3.24, 95% CI: 2.58, 3.99; *β*_Age_ = −0.52, 95% CI: −0.91, −0.14; Figure S3). However, there was no evidence that food availability at birth influenced this trend (Table 2, Figure 4a). A negative effect of AFR was observed (*β*_AFR_ = −0.72, 95% CI: −1.22, −0.42), while no significant effects were found for ALR (*β*_ALR_ = 0.14, 95% CI: −0.42, 0.68), site (*β*_[site = Vaasa]_ = −0.14, 95% CI: −0.99, 0.71), or age at marking (*β*_MarkingAge_ = −0.09, 95% CI: −0.96, 0.76).

Instead, we observed a decline in litter size with age with a breakpoint at 5 years old (Table 1; Figure 4b; *β*_0_ = 0.94, 95% CI: 0.84, 1.03, *β*_age.pre‐onset_ = −0.02, 95% CI: −0.07, 0.02; *β*_age. post‐onset_ = −1.76, 95% CI: −4.51, −0.39 from M1_d_ Table 2). An additive effect of birth food availability on litter size was detected (Table 2; *β*_BirthFood_ = −0.06, 95% CI: −0.10, −0.01), with mothers born under unfavourable food conditions tending to have slightly larger litters. No effect of AFR (*β*_AFR_ = −0.03, 95% CI: −0.07, 0.01), ALR (*β*_ALR_ = 0.02, 95% CI: −0.02, 0.06), age at marking (*β*_MarkingAge_ = −0.003, 95% CI: −0.06, 0.06) or site (*β*_[site = Vaasa]_ = −0.002, 95% CI: −0.06, 0.06) were detected. The mean litter size in our study areas was 1.53 [SD = 1.32] juveniles, increasing to 2.44 [0.75] when considering only litters greater than 0.

Maternal age also explained some variation in juvenile body mass. The best supported model included maternal age as a quadratic term (Table 1; *β*_0_ = 59.46, 95% CI: 57.20, 61.76; *β*_Age_ = 1.20, 95% CI: 0.51, 1.88; *β*_Age2_ = −0.97, 95% CI: −1.30, −0.65). However, age‐related changes in body mass were weak and not influenced by early life food availability experienced by the mothers (Table 2; Figure 4c). ALR (*β*_ALR_ = 1.10, 95% CI: 0.18, 2.04) and age at marking (*β*_MarkingAge_ = 1.61, 95% CI: 0.13, 3.13) showed positive trends, while AFR had no effect (*β*_AFR_ = 0.57, 95% CI: −0.12, 1.27). Juveniles at Vaasa were on average lighter (mean [SD] = 56.69 g [10.51]) than juveniles at Luoto (mean [SD] = 59.64 g [9.02]; *β*_[site = Vaasa]_ = −3.73, 95% CI: −5.21, −2.22).

While we found a negative quadratic relationship between juvenile body mass and litter size, revealing a size‐number trade‐off, we did not detect a significant increase in the strength of this trade‐off with increasing maternal age (pairwise comparisons, *p*‐values > 0.05; slopes from *β*_[age4]_ = −4.82, 95% CI [−7.52, −2.11] to *β*_[age3]_ = −8.79, 95% CI [−10.45, −7.14]) and/or when mothers experienced lower resources at birth (Tables 1 and 2, Figure S6).

## 4 DISCUSSION

We characterised senescence patterns and tested whether early life food availability from tree masting shaped age‐specific variations across a comprehensive range of demographic and morphological traits in two wild populations of Siberian flying squirrels. Our results provide new evidence of survival senescence from the age of 3 years, a moderate senescence of maternal body mass and two reproductive traits, the annual reproductive probability and litter size, starting at 4–5 years old. Despite a weak, positive effect of early life resources on adult female survival, we found no evidence supporting the disposable soma theory, the internal predictive adaptive response or the silver‐spoon hypothesis. Experiencing a low or high food availability at birth did not influence the rate of senescence in survival, morphological and reproductive traits. Our results suggest that food availability at birth had a slight effect on life history traits at adulthood, but no effect on senescence patterns in this fast‐living mammal.

### 4.1 Demographic and morphological senescence in a short‐lived mammal

Survival and reproductive senescence are ubiquitous in the wild, although there is considerable variation among species and individuals. Local environmental conditions (Cooper & Kruuk, 2018; Sanghvi et al., 2022), mean body size, pace of life and phylogeny (Jones et al., 2008; Lemaître et al., 2020) have all been identified as key factors shaping this variation. Given the relatively fast life history of the Siberian flying squirrel, we expected an early onset at the age of first reproduction (i.e. second calendar year of life) and a fast rate of demographic and morphological senescence (if detected) in both study populations.

Our results gave support for a fast rate of survival senescence among female flying squirrels but at a later onset than expected, from the age of 3 years onwards. In contrast, patterns of reproductive senescence, if detected, started at the age of 4 years (litter size and annual reproductive probability). We did not detect an effect of ageing on the offspring size‐number trade‐off or on juvenile body mass. However, these results may be influenced by uncertainties in body mass estimates, since juveniles were weighed at different times between birth and weaning.

Overall, our findings support the view that reproductive (Lemaître et al., 2020) and survival senescence (Nussey et al., 2013; Shefferson et al., 2017) are common in mammals, although it has primarily been evidenced in long‐lived or larger‐sized mammals and captive small mammals. Senescence patterns showed consistent onsets, indicating a general decline in organismal function starting at 3 to 4 years old. Nevertheless, reproductive senescence is not widespread across all traits studied, as reported in previous studies on other fast‐living sciurids. Richardson's ground squirrel (*Spermophilus richardsonii*), with a maximum lifespan of 6 years, showed survival senescence and no decline in reproductive traits with age (Broussard et al., 2005). While both survival and reproductive senescence were detected in the American red squirrel (*Tamiasciurus hudsonicus*), the latter was weaker and only observed on certain traits (Descamps et al., 2008). A late onset of senescence was also identified in this species (between 4 to 6 years old, while maturity is reached at 1 year of age and maximum lifespan is 9 years). In contrast, among longer‐lived rodents (lifespan >9 years), such as the Columbian ground squirrel (*Spermophilus columbianus*; Broussard et al., 2003) and the Alpine marmot (*Marmota marmota*; Berger et al., 2015), a strong senescence of reproductive traits was observed.

The variation in senescence patterns among reproductive traits in our study illustrates the complexity of studying reproductive senescence in the wild, when heterogeneous rates of senescence may occur for different reproductive components (Lemaître & Gaillard, 2017). Reproductive senescence can derive from a decrease of maternal performance in different components (e.g. fecundity, offspring size‐number trade‐off, reproductive effort) and/or from maternal effect senescence when increased maternal age has a detrimental effect on offspring performance (e.g. offspring body mass, survival and other fitness traits; Moorad & Nussey, 2016). For instance, juvenile survival but not juvenile body mass of North American red squirrels declined with increasing maternal age (Descamps et al., 2008). In our study populations, older mothers tended to produce juveniles of lower mass, suggesting that these juveniles may be of lower quality. However, due to strong natal dispersal and limited sample size, the relationship between maternal age and survival and reproductive success of their juveniles is unknown and should be further investigated to fully understand maternal effect senescence in flying squirrels.

Body mass is a key trait associated with organismal functioning (e.g. physical and fat conditions), reproduction and survival. Empirically, sciurids show contrasted evidence of body mass senescence in females (e.g. evidence: Broussard et al., 2003; Kroeger et al., 2018; no detection: Descamps et al., 2008; Tafani et al., 2013). In flying squirrels, we found a slight decline in maternal body mass with age in females aged 4 years and older (quadratic and threshold models selected). This weak body mass senescence might be explained by specific optimization trade‐offs that vary with environmental conditions (Stearns, 1992). In years of low food availability during adulthood, females could compromise their reproduction to maintain their condition as found in other sciurids (e.g. Kroeger et al., 2018), although the existence of a trade‐off between reproduction and body condition remains to be tested. Alternatively, weak body mass senescence might also be a consequence of the flying squirrel's income‐capital breeding strategy. Flying squirrels are categorised as capital breeders (Selonen & Wistbacka, 2016; i.e. reproduction based on stored resources from body‐fat reserves and/or food hoarding). However, the income‐capital breeding strategy can vary throughout the breeding seasons according to the availability and predictability of resources, and the individual condition (Williams et al., 2017). For instance, Siberian chipmunks exhibit an income breeder strategy in summer, when numerous and diverse resources are available, and a partial capital breeder strategy in spring, relying on food hoarded prior to hibernation (Le Coeur et al., 2018). Such flexibility in income‐capital breeding strategy may also occur in flying squirrels, where reproductive events are based on catkin storage and possibly influenced by concurrent food intake. Finally, we cannot exclude that body mass changes with age were mostly masked because females accumulated enough weight during spring before being weighed in June.

Despite evidence of an offspring size‐number trade‐off in our populations, we did not find evidence of a linear, negative trend in age‐specific changes in this trade‐off. This result is consistent with previous studies investigating the influence of age on this trade‐off in Alpine marmots (Berger et al., 2015) and Soay sheep (Hayward et al., 2013). Similar to marmots, litter size in flying squirrels declines with maternal age, while juvenile body mass decreases only slightly. Consequently, older flying squirrels tend to maximise their reproductive success by maintaining juvenile quality. Lemaître and Gaillard (2017) argued that this pattern, underlying the lack of an age effect on the size‐number trade‐off, may be related to environmental predictability. Flying squirrel reproduction in spring is highly dependent on cached winter food (catkins) and influenced by weather conditions in spring and the preceding winter (Selonen et al., 2016; Selonen & Wistbacka, 2016), resulting in relatively predictable environmental conditions for reproduction. The size‐number trade‐off observed in flying squirrels may therefore represent an adaptation to these predictable, yet highly negatively autocorrelated, food conditions encountered at birth and throughout adulthood.

### 4.2 No evidence for effects of early life conditions on senescence patterns

The early stages of life are critical periods for an individual, when available resources are allocated between growth, first reproductive events and somatic maintenance. The environmental conditions experienced during these stages cannot only affect fitness‐related traits at a specific time in adult life but also potentially how these traits change with age. Evolutionary hypotheses of senescence predict that lower/higher rates of senescence related to early environmental conditions could arise from early late life trade‐offs (disposable soma theory) or lifelong effects of unfavourable (iPAR) and favourable natal conditions (silver spoon effect).

Contrary to our expectations, food conditions at birth did not significantly affect senescence patterns in reproductive and morphological traits. Interestingly, females that experienced low food availability during their first year produced more juveniles in their second year of life compared to those exposed to catkin mast events at birth. Whether this increased juvenile production at sexual maturity led to accelerated or more pronounced senescence would support the disposable soma theory or iPAR. Unfortunately, the small sample size of senescent females prevented us from testing these hypotheses on litter size. In contrary, females tend to survive better at adulthood when they experienced abundant food during early life, which may align with the silver spoon hypothesis. However, no significant long‐lasting effects or changes on senescence rate were detected.

The lack of long‐lasting effects of birth food resources on demographic and morphological traits may be the result of strong negative temporal autocorrelation of alder and birch catkin production at our study sites, which leads to a succession of years with good and poor catkin production, preventing two successive mast events. Alder and birch catkin production at a given point or cumulative availability of food during adulthood may buffer the positive or negative impact of early life conditions on performance and senescence patterns (Sanghvi et al., 2022). Strong silver‐spoon effects on senescence are thus unlikely to be detected in this food pulse system (Cooper & Kruuk, 2018), nor are they a fitness benefit of matching environments between early life and specific stages of adulthood, as suggested by the external predictive adaptive response hypothesis (Nettle et al., 2013). A similar dynamic was observed in North American red squirrels, where the negative effects of early life conditions on adult lifespan were buffered by a spruce mast event in their second year of life (Petrullo et al., 2024).

Limited long‐lasting effects of pulsed resource early in life on late life performance might also be attributed to concomitant early life stressors, such as the annual density of predators (Petrullo et al., 2024; Selonen & Mäkeläinen, 2017) that may alter effects expected from resource condition alone. It can also be linked to immediate and substantial mortality during development due to strong selection early in life. As expected in a fast‐living species, survival of juveniles (0.15–0.20; Brommer et al., 2017) and young adult females reaching 2 years of age (*Phi*_Age1‐2_ = 0.50 ± 0.03) was particularly low in the study populations, thus many individuals, especially those born in poor conditions, may not survive long enough to express senescence.

In conclusion, this study provides new evidence for survival, reproductive and body mass senescence in wild populations of a fast‐living rodent, the Siberian flying squirrel. However, we did not detect any effect of natal resource conditions, measured by the highly variable production of catkins from alder and birch masting, on late‐life performance.

## AUTHOR CONTRIBUTIONS

Christie Le Coeur, Vérane Berger and Vesa Selonen conceived the ideas and designed methodology; Ralf Wistbacka collected the data; Christie Le Coeur and Vérane Berger analysed the data, Christie Le Coeur drafted the manuscript, with input from all authors. Our study brings together authors from different countries, including scientists based in the country where the study was carried out. Whenever relevant, literature published by local researchers was cited.

## CONFLICT OF INTEREST STATEMENT

The authors declare no conflict of interest.

## Supporting information


**Figure S1.** A. Annual variation in the two landscape‐level food availability estimates, alder pollen (accumulated sums of average daily counts of airborne pollen/m^3^ during spring) and birch catkins (average catkins per tree counted in the previous winter) over 24 years, 1991–2014.
**Figure S2.** Relationship between maternal body mass (in grams), age and food availability at birth.
**Figure S3.** Variation in female annual reproductive probability according to age (in years) and food availability experienced at birth).
**Figure S4.** Relationship between juvenile body mass and day of body mass measurement within the breeding season.
**Figure S5.** Variation in juvenile body mass before weaning (in grams) according to maternal age (in years) and food availability experienced by the mothers at birth.
**Figure S6.** Variation in juvenile body mass–litter size trade‐off according to maternal age (in years).
**Figure S7.** Age‐dependent estimates of annual female survival probabilities (circles; 95% confidence intervals are indicated by vertical bars) according to age at marking (marked as juvenile or adult in red and blue, respectively; estimates from model *phi*(age + MarkingAge)p(.) with age a categorical variable).
**Table S1.** AIC model selection table for maternal body mass.
**Table S2.** Model selection table for female annual reproductive probability.
**Table S3.** AIC model selection for litter size.
**Table S4.** AIC model selection table for the influence of maternal age on juvenile body mass.
**Table S5.** AIC model selection table for the influence of maternal age on juvenile body mass, when females of 5 and 6 years of age (*n* = 8 and 2, respectively) were grouped together.
**Table S6.** AIC model selection for age‐specific variation of the juvenile body mass‐number trade‐off.
**Table S7.** Goodness of fit tests for trap‐dependence and transience effect at each site (Vaasa and Luoto).
**Table S8.** Results of the selection procedure on recapture (*p*) parameters using age as a categorical variable or continuous variable (logit and loglog link).
**Table S9.** AIC model selection for age‐specific variation in survival (*phi*). Age at marking, site and annual food availability at adulthood were included as covariates.
**Table S10.** AIC model selection for *phi* using ‘time’ (years of monitoring from 1992 to 2014) as a covariate.

## Data Availability

Data available from the Dryad Digital Repository: https://doi.org/10.5061/dryad.7d7wm384j (Le Coeur et al., 2024).
