# Supplementary material for: No evidence of early life resource pulse effects on age‐specific variation in survival, reproduction and body mass of female Siberian flying squirrels
Source: J Anim Ecol. 2024 Nov 11;93(12):2024–37. doi: 10.1111/1365-2656.14218 (PMC11615270; doi:10.1111/1365-2656.14218)
Supplement: Supplementary file 1 — Figure S1. A. Annual variation in the two landscape‐level food availability estimates, alder pollen (accumulated sums of average daily counts of airborne pollen/m3 during spring) and birch catkins (average catkins per tree counted in the previous winter) over 24 years, 1991–2014. Figure S2. Relationship between maternal body mass (in grams), age and food availability at birth. Figure S3. Variation in female annual reproductive probability according to age (in years) and food availability experienced at birth). Figure S4. Relationship between juvenile body mass and day of body mass measurement within the breeding season. Figure S5. Variation in juvenile body mass before weaning (in grams) according to maternal age (in years) and food availability experienced by the mothers at birth. Figure S6. Variation in juvenile body mass–litter size trade‐off according to maternal age (in years). Figure S7. Age‐dependent estimates of annual female survival probabilities (circles; 95% confidence intervals are indicated by vertical bars) according to age at marking (marked as juvenile or adult in red and blue, respectively; estimates from model phi(age + MarkingAge)p(.) with age a categorical variable). Table S1. AIC model selection table for maternal body mass. Table S2. Model selection table for female annual reproductive probability. Table S3. AIC model selection for litter size. Table S4. AIC model selection table for the influence of maternal age on juvenile body mass. Table S5. AIC model selection table for the influence of maternal age on juvenile body mass, when females of 5 and 6 years of age (n = 8 and 2, respectively) were grouped together. Table S6. AIC model selection for age‐specific variation of the juvenile body mass‐number trade‐off. Table S7. Goodness of fit tests for trap‐dependence and transience effect at each site (Vaasa and Luoto). Table S8. Results of the selection procedure on recapture (p) parameters using age as a categorical variable or continuous variab [file JANE-93-2024-s001.pdf]

Supporting information - AIC model selection tables for early-life pulsed resource effects on age-specific variation in maternal body mass, annual reproductive probability, litter size, juvenile body mass, juvenile size-number trade-off and maternal survival in two Finnish populations of Siberian flying squirrels.

Early-life pulsed resource from birch and alder masting - To investigate the influence of food availability at birth on age-specific variation and senescence patterns, we used both alder and birch availability in spring per year. Birch catkin production was sampled annually in winter at Vaasa by the Finnish Forest Research Institute (Hokkanen, 2000). Although the birch availability index is less accurate for Luoto than for Vaasa, this index properly describes the yearly variation in catkin production in this area due to very similar weather conditions between the two coastal sites and high spatial correlation in birch catkin production at scales of up to several hundred kilometres in Finland (Gallego-Zamorano, Hokkanen, & Lehikoinen, 2016; Ranta et al., 2008). As a proxy of alder catkin production for both sites, we used aerial pollen data sampled in Vaasa by the aerobiology unit at the University of Turku (Ranta et al., 2008). The data consisted of accumulated sums of average daily counts of airborne pollen/m^3 of air during spring (see Fig. 1 in the main body of the manuscript). After scaling the annual estimates of alder and birch resource availability separately, we calculated the average of the two scaled variables per year. The annual variation of this composite variable is shown below (Fig. S1).

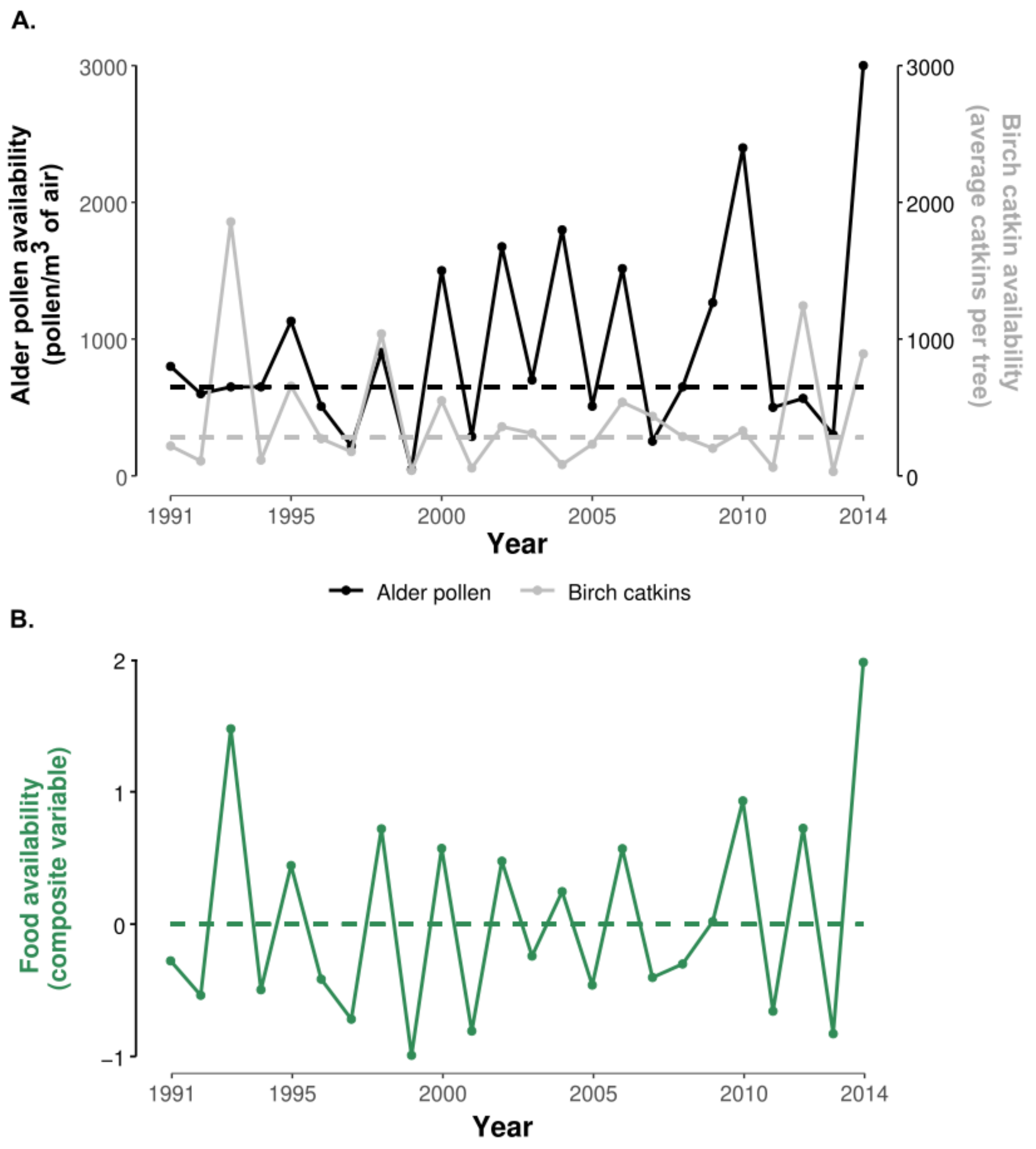

Figure S1. A. Annual variation in the two landscape-level food availability estimates, alder pollen (accumulated sums of average daily counts of airborne pollen/m^3 during spring; black line) and birch catkins (average catkins per tree counted in the previous winter; grey line) over 24 years, 1991-2014. Black and grey dashed lines refer to the median value of alder pollen availability and birch catkin availability, respectively. B. Annual variation in the composite variable (mean values of scaled alder and birch resource availability).

1) Model selection for maternal body mass

Maternal body mass was analysed using linear mixed models (*lme4 R* package), including female ID, birth year, year of observation and forest patch (local spatial heterogeneity) as crossed random factors. First, we ran a set of candidate models that included age as a linear, quadratic or threshold term (from age 2 to age 5), and compared the AICc between models. AFR and ALR, the age at which a female was first and last known to reproduce, were included as covariates in the models to assess the relative contribution of selective appearance and disappearance, respectively. Site, reproductive month (*PregnancyClass - see definition below*), age at marking (*MarkingAge*) and adult food availability in year *t* (*AnnualFood*) were added as covariates in the models. Second, from the best AICc model(s), we tested for the disposable soma theory and silver-spoon hypothesis by examining the additive effect and two-way interaction between birth food availability and age (Table S1).

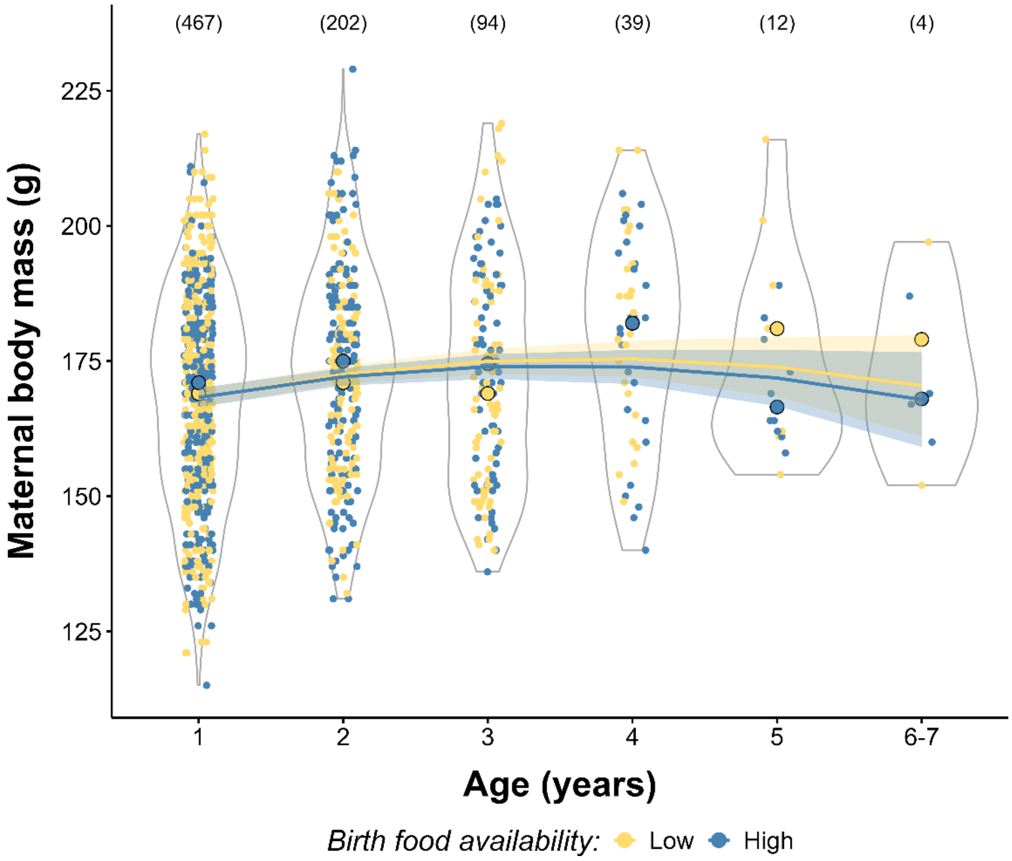

Figure S2. Relationship between maternal body mass (in grams), age and food availability at birth (below and above the median in yellow and blue, respectively). Lines and shades correspond to the prediction of the quadratic model (~Age * BirthFood + Age^2 in Table S1) and the 95% confidence intervals, respectively. Violins give the distribution of the observed values for each age. Yellow and blue dots with black outlines indicate maternal body mass median at each age for each food availability category. The number of unique individuals for each age is shown at the top.

Table S1. AIC model selection table for maternal body mass. Site, AFR, ALR, PregnancyClass age at marking and annual food availability were included in the models as covariates. The number of parameters (np), the Akaike's Information Criterion corrected for small sample sizes (AICc), ΔAICc, model weight (ω) and deviance are presented.

Model structure	np	AICc	ΔAICc	ω	Deviance
a) Selection on age					
**Quadratic Age**	**14**	**9404.41**	**0.00**	**0.47**	**9376.02**
**Threshold_Age4**	**14**	**9405.23**	**0.82**	**0.31**	**9376.84**
Threshold_Age2	14	9406.41	2.00	0.17	9378.02
Threshold_Age3	14	9409.62	5.21	0.03	9381.23
Threshold_Age5	14	9411.25	6.84	0.02	9382.86
Linear	13	9414.63	10.22	0.00	9388.29
Constant	12	9447.70	43.29	0.00	9423.42
b) Effect of birth food availability (from the quadratic model)					
**Age + Age^2**	**14**	**9404.41**	**0.00**	**0.39**	**9376.02**
**Age * BirthFood + Age^2**	**16**	**9405.61**	**1.20**	**0.21**	**9373.11**
**Age + BirthFood * Age^2**	**16**	**9405.96**	**1.55**	**0.18**	**9373.46**
Age + Age^2 + BirthFood	15	9406.46	2.05	0.14	9376.02
(Age + Age^2) * BirthFood	17	9407.65	3.24	0.08	9373.08
c) Effect of birth food availability (from threshold model 4: $Age_{pre\text{-}onset}$ + $Age_{post\text{-}onset}$) $Age_{pre\text{-}onset}$ = age ≤4 and $Age_{post\text{-}onset}$ = age >4					
**$Age_{pre\text{-}onset}$ + $Age_{post\text{-}onset}$**	**14**	**9405.23**	**0.00**	**0.45**	**9376.84**
**$Age_{pre\text{-}onset}$ * BirthFood + $Age_{post\text{-}onset}$**	**16**	**9406.61**	**1.38**	**0.23**	**9374.10**
$Age_{pre\text{-}onset}$ + $Age_{post\text{-}onset}$ + BirthFood	15	9407.26	2.03	0.16	9376.81
($Age_{pre\text{-}onset}$ + $Age_{post\text{-}onset}$) * BirthFood	17	9408.67	3.44	0.08	9374.10
$Age_{pre\text{-}onset}$ + BirthFood * $Age_{post\text{-}onset}$	16	9408.95	3.72	0.07	9376.45

Abbreviations: BirthFood = food availability at birth; MarkingAge = marked as juveniles or as adults (yearlings); pregnancyClass = reproductive month (2-level factor with "1" for June and August. 0 otherwise); site = Vaasa and Luoto; Threshold_AgeX = threshold model with a breakpoint at age X ($Age_{pre\text{-}onset}$ and $Age_{post\text{-}onset}$ correspond to age before and after the onset of senescence).

2) Model selection for female annual reproductive probability

To analyse the female annual reproductive probability, we used generalised linear mixed models with a binomial distribution (*glmmTMB* package). To avoid convergence problems, only Female ID and year of observation were treated as random factors. First, we ran a set of candidate models that included age as a linear, quadratic or threshold term (from age 2 to age 5), and compared the AICc between models. AFR and ALR (age of first and last reproduction), site, age at marking (*MarkingAge*) and adult food availability in year *t* (*AnnualFood*) were added as covariates in the models. Second, from the best AICc model(s), we tested for the disposable soma theory and silver-spoon hypothesis by examining the additive effect and two-way interaction between birth food availability and age (Table S2).

Table S2. Model selection table for female annual reproductive probability. Site, AFR, ALR, age at marking (*MarkingAge*) and annual food availability (*AnnualFood*) were included in the models as covariates. The number of parameters (np), the Akaike's Information Criterion corrected for small sample sizes (AICc), ΔAICc, model weight (ω) and deviance are presented.

**Model structure**	**np**	**AICc**	**ΔAICc**	**ω**	**Deviance**
a) Selection on age					
**Threshold_Age5**	**10**	**313.24**	**0.00**	**0.29**	**287.97**
**Threshold_Age4**	**10**	**313.36**	**0.12**	**0.27**	**289.54**
**Linear**	**9**	**314.31**	**1.07**	**0.17**	**295.50**
Threshold_Age2	10	315.39	2.15	0.10	241.97
Quadratic Age	10	316.07	2.83	0.07	294.50
Threshold_Age3	10	316.22	2.98	0.06	295.94
Constant	8	316.95	3.71	0.04	194.46
b) Effect of birth food availability (from the linear model)					
**Age**	**9**	**314.31**	**0.00**	**0.39**	**295.50**
**Age + BirthFood**	**10**	**314.41**	**0.10**	**0.37**	**294.12**
**Age * BirthFood**	**11**	**315.36**	**1.05**	**0.23**	**293.02**
c) Effect of birth food availability (from Threshold model 4: $Age_{pre-onset}$ + $Age_{post-onset}$) $Age_{pre-onset}$ = age ≤4 and $Age_{post-onset}$ = age >4					
**$Age_{pre-onset}$ + $Age_{post-onset}$**	**10**	**313.36**	**0.00**	**0.34**	**289.54**
**$Age_{pre-onset}$ + $Age_{post-onset}$ + BirthFood**	**11**	**313.50**	**0.14**	**0.31**	**290.02**
**$Age_{pre-onset}$ * BirthFood + $Age_{post-onset}$**	**12**	**314.88**	**1.52**	**0.16**	**290.48**
**$Age_{pre-onset}$ + $Age_{post-onset}$ * BirthFood**	**12**	**315.19**	**1.83**	**0.13**	**290.44**
($Age_{pre-onset}$ + $Age_{post-onset}$) * BirthFood	13	316.91	3.55	0.06	290.44

Abbreviations: BirthFood = food availability at birth; Threshold_AgeX = threshold model with a breakpoint at age X ($Age_{pre-onset}$ and $Age_{post-onset}$ correspond to age before and after the onset of senescence).

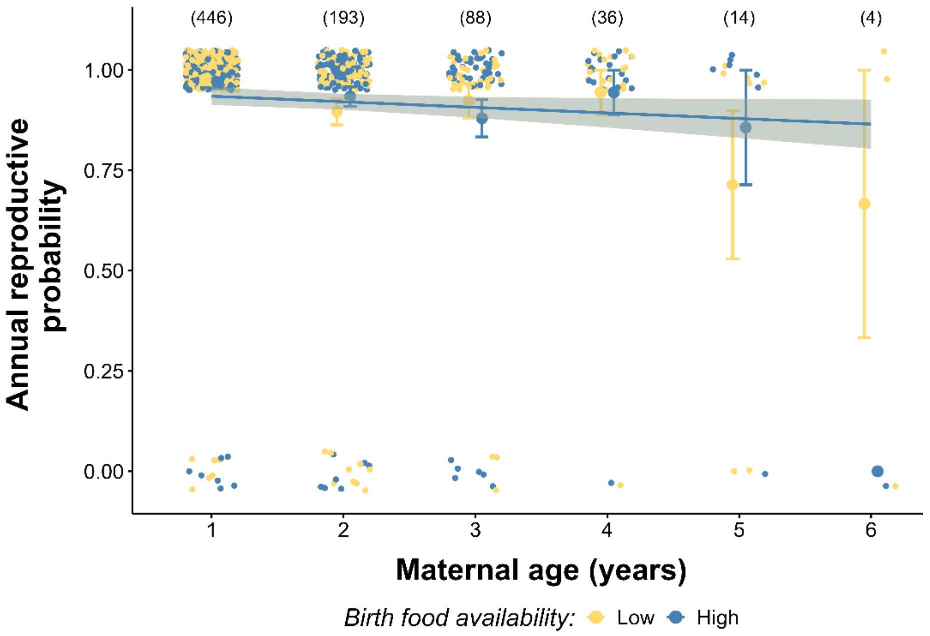

Figure S3. Variation in female annual reproductive probability according to age (in years) and food availability experienced at birth (below and above the median in yellow and blue, respectively). Lines and shades correspond to the prediction of the ***linear model 'Age + BirthFood'*** (Table S2) and the 95% confidence intervals, respectively. Mean values are shown as circles ± standard errors. Observed values (0 or 1) at each age for each food availability category are represented by yellow and blue dots.

3) Model selection for litter size of female Siberian flying squirrels

Zero-inflated generalized linear mixed models (*glmmTMB* R package) with a Conway–Maxwell Poisson distribution were applied to investigate the age-specific variation in litter size of female flying squirrels. Female ID, year of observation and forest patch (local spatial heterogeneity) were treated as crossed random factors. Modelling fit was assessed with residual diagnostics (*DHARMa* R package). As with the other traits, we first ran and compared by AICc a set of candidate models that included age as a linear, quadratic or threshold term (from age 2 to age 5). AFR and ALR (age of first and last reproduction), site, age at marking (*MarkingAge*) and adult food availability in year *t* (*AnnualFood*) were added as covariates in the models. Second, from the best AICc model(s), we tested for the disposable soma theory and silver-spoon hypothesis by examining the additive effect and two-way interaction between birth food availability and age. Litter size was defined as the number of juveniles per litter, ranging from 0 to 4+ (with one record of five juveniles), only among females observed in the nest during the first reproductive season.

Table S3. AIC model selection for litter size. Site, AFR, ALR, age at marking (*MarkingAge*) and annual food availability (*AnnualFood*) were included in the models as covariates. The number of parameters (np), the Akaike's Information Criterion corrected for small sample sizes (AICc), ΔAICc, model weight (ω) and deviance are presented.

**Model structure**	**np**	**AICc**	**ΔAICc**	**ω**	**Deviance**
a) Selection on age					
**Threshold_Age5**	**13**	**2169.11**	**0.00**	**0.59**	-
Threshold_Age3	13	2172.45	3.34	0.11	-
Linear	12	2173.27	4.16	0.07	-
Threshold_Age4	13	2173.30	4.19	0.07	-
Quadratic Age	13	2173.55	4.44	0.06	-
Constant	11	2173.64	4.53	0.06	-
Threshold_Age2	13	2175.11	6.00	0.03	-
b) Effect of birth food availability (from the best threshold model at Age5) $Age_{pre\text{-}onset}$ = age ≤5 and $Age_{post\text{-}onset}$ = age >5					
**$Age_{pre\text{-}onset}$ + $Age_{post\text{-}onset}$ + BirthFood**	**14**	**2165.24**	**0.00**	**0.66**	-
$Age_{pre\text{-}onset}$ * BirthFood + $Age_{post\text{-}onset}$	15	2167.24	2.00	0.24	-
$Age_{pre\text{-}onset}$ + $Age_{post\text{-}onset}$	13	2169.11	3.87	0.10	-

Abbreviations: BirthFood = food availability at birth; Threshold_AgeX = threshold model with a breakpoint at age X ($Age_{pre\text{-}onset}$ and $Age_{post\text{-}onset}$ correspond to age before and after the onset of senescence).

4) Influence of maternal age on juvenile body mass before weaning

We used linear mixed models (*nlme* package) to examine the senescence of body mass in juveniles, produced during the first breeding season of the year, as a function of maternal age. Mother ID, forest patch (local spatial heterogeneity) and year of observation were treated as random factors. Site, litter size (number of juveniles in the litter), day of body mass measurement ('DaySinceJan1'), age at marking, and adult food availability in year *t* were added as covariates in the models. Day of measurement was used to account for intra-annual variation in the date of birth and date of body mass measurement among juveniles (Fig. S4). Body mass measurements between 1992 and 2014 started on average on June 4 (May 15-June 9 depending on the year) and ended on June 22 (June13-July 5 depending on the year).

In a second step, the additive effect of food availability encountered by the mothers at birth and the interaction between birth food availability and maternal age on juvenile body mass were tested (Table S4). We ran the same analysis by grouping females of 5 and 6 years of age (n= 8 and 2, respectively; Table S5 and Fig. S5).

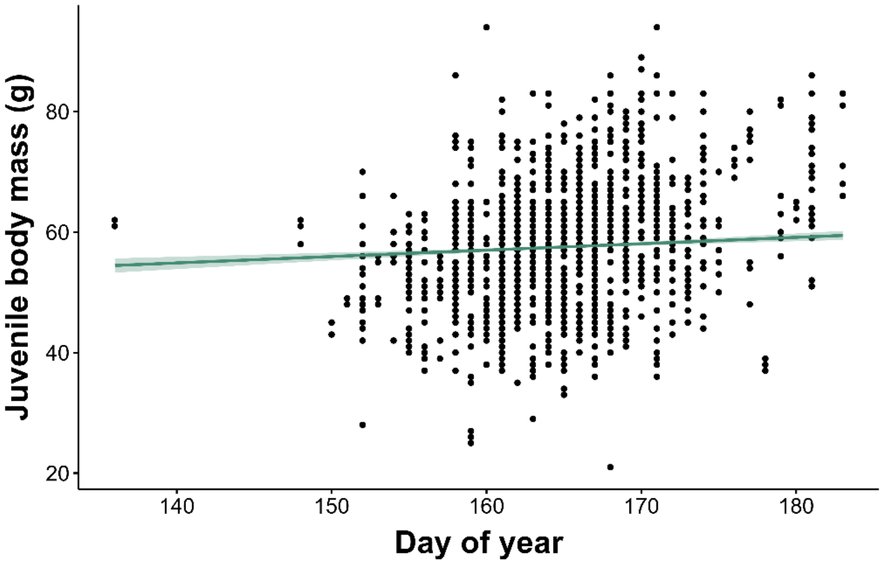

Figure S4. Relationship between juvenile body mass and day of body mass measurement within the breeding season. All measurements are shown (circles) and the green line shows predicted body mass with associated 95% CI, adjusted for all other covariates in model 'Quadratic Age' (±95% CI; Table S5).

Table S4. AIC model selection table for the influence of maternal age on juvenile body mass. Site, AFR, ALR, DaySinceJan1, age at marking, and annual food availability were included in the models as covariates. Mother ID, forest patch and year of observation were treated as random factors. The number of parameters (np), the Akaike's Information Criterion corrected for small sample sizes (AICc), ΔAICc, model weight (ω) and deviance are presented.

**Model structure**	**np**	**AICc**	**ΔAICc**	**ω**	**Deviance**
a) Selection on age					
**Quadratic Age**	**14**	**10176.24**	**0.00**	**0.95**	**10147.95**
Threshold_Age2	14	10182.71	6.47	0.04	10154.43
Threshold_Age4	14	10186.61	10.37	0.01	10158.32
Threshold_Age3	14	10188.18	11.94	0.00	10159.90
Threshold_Age5	14	10207.18	30.94	0.00	10178.90
Constant	12	10208.69	32.45	0.00	10184.48
Linear	13	10209.04	32.80	0.00	10182.80
b) Effect of food availability experienced by the mothers at birth (from the quadratic model)					
**Age + Age^2**	**14**	**10176.24**	**0.00**	**0.52**	**10147.95**
**Age + Age^2 + BirthFood**	**15**	**10178.07**	**1.83**	**0.21**	**10147.75**
Age * BirthFood + Age^2	16	10179.15	2.91	0.12	10146.79
Age + BirthFood * Age^2	16	10179.25	3.01	0.11	10146.89
(Age + Age^2) * BirthFood	17	10181.19	4.95	0.04	10146.78

Table S5. AIC model selection table for the influence of maternal age on juvenile body mass, when females of 5 and 6 years of age (n= 8 and 2, respectively) were grouped together. Site, AFR, ALR, DaySinceJan1 and annual food availability were included in the models as covariates. The number of parameters (np), the Akaike's Information Criterion corrected for small sample sizes (AICc), ΔAICc, model weight (ω) and deviance are presented.

**Model structure**	**np**	**AICc**	**ΔAICc**	**ω**	**Deviance**
a) Selection on age					
**Quadratic Age**	**14**	**10176.64**	**0.00**	**0.96**	**10148.36**
Threshold_Age2	14	10183.80	7.16	0.03	10155.52
Threshold_Age4	14	10185.98	9.34	0.01	10157.70
Threshold_Age3	14	10189.43	12.79	0.00	10161.15
Constant	12	10208.69	32.05	0.00	10184.48
Linear	13	10209.34	32.70	0.00	10183.10
b) Effect of birth food availability (from the quadratic model Age + Age^2)					
**Age + Age^2**	14	10176.64	0.00	0.50	10148.36
**Age + Age^2 + BirthFood**	15	10178.47	1.83	0.20	10148.15
Age * BirthFood + Age^2	16	10179.37	2.73	0.13	10147.00
Age + BirthFood * Age^2	16	10179.43	2.79	0.12	10147.07
(Age + Age^2) * BirthFood	17	10181.41	4.77	0.05	10147.00

Abbreviations: AFR = Age of first reproduction; ALR = age of last reproduction; BirthFood = food availability at birth; site = Vaasa and Luoto; Threshold_AgeX = threshold model with a breakpoint at age X ($Age_{pre\text{-}onset}$ and $Age_{post\text{-}onset}$ correspond to age before and after the onset of senescence).

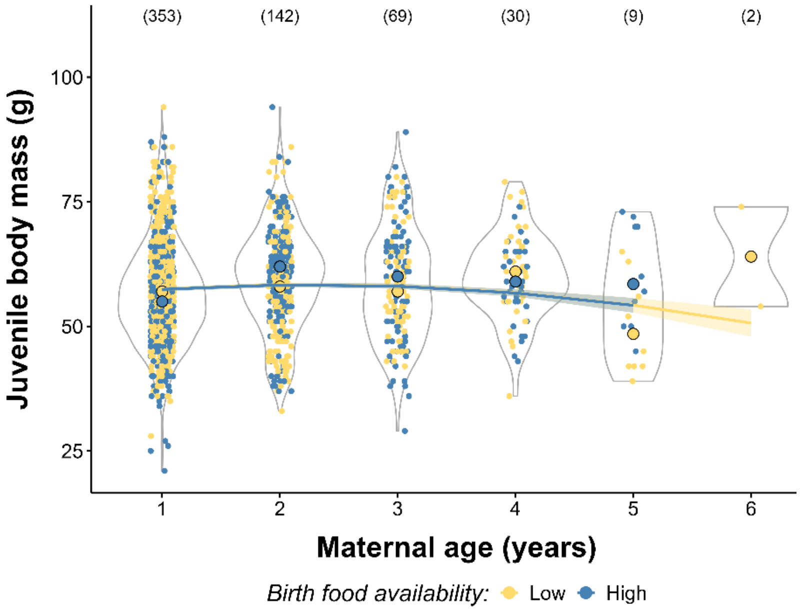

Figure S5. Variation in juvenile body mass before weaning (in grams) according to maternal age (in years) and food availability experienced by the mothers at birth (below and above the median in yellow and blue, respectively). Females of age 5 and 6 were not grouped together. Lines and shades correspond to the prediction of the ***quadratic model 'Age + Age²* + *BirthFood'*** (Table S5) and the 95% confidence intervals, respectively. Violins give the distribution of the observed values for each age. Yellow and blue dots with black outlines indicate the median juvenile body mass at each age for each food availability category.

5) Juvenile size-number trade-off

We tested whether the trade-off between juvenile body mass and the number of juveniles produced in a litter is affected by maternal age (linear effect of age only) and food availability experienced by the mothers in early life. Juvenile body mass was the variable of interest. Mother ID, forest patch and year of observation were treated as random factors in linear mixed models. Site, age at first reproduction (AFR), age at last reproduction (ALR), Day of body mass measurement (DaySinceJan1) and annual food availability were included in the models as covariates.

Table S6. AIC model selection for age-specific variation of the juvenile body mass-number trade-off. Site, AFR, ALR, DaySinceJan1, age of mothers at marking, and annual food availability were included in the models as covariates. The number of parameters (np), the Akaike's Information Criterion corrected for small sample sizes (AICc), ΔAICc, model weight (ω) and deviance are presented.

Model structure	np	AICc	ΔAICc	ω	Deviance
a) Effect of maternal age on the juvenile size-number trade-off					
**Age * N_{juv} + N_{juv}^2**	**14**	**10213.52**	**0.00**	**0.46**	**10185.24**
**N_{juv} + N_{juv}^2 * Age**	**14**	**10214.4**	**0.88**	**0.30**	**10186.12**
**Age * (N_{juv} + N_{juv}^2)**	**15**	**10215.26**	**1.74**	**0.19**	**10184.94**
Age + (N_{juv} + N_{juv}^2)	13	10219.53	6.01	0.02	10193.28
Age * N_{juv}	13	10220.28	6.76	0.02	10194.03
Age + N_{juv}	12	10223.88	10.36	0.00	10199.67
b) Effect of food availability experienced by the mothers at birth					
**Age * N_{juv} + N_{juv}^2**	**14**	**10215.65**	**0.00**	**0.68**	**10187.37**
**Age * N_{juv} + N_{juv}^2 + BirthFood**	**15**	**10217.59**	**1.94**	**0.26**	**10187.27**
Age * N_{juv} * BirthFood + N_{juv}^2	18	10220.54	4.89	0.06	10184.08

Abbreviations: Age = linear effect of Age; BirthFood = food availability experienced by the mothers at birth; N_{juv} = litter size.

From the top model 'Age * N_{juv} + N_{juv}^2', the estimated slopes of N_{juv} for each maternal age category were as follows:

$\beta_{[\text{Maternal age 1}]}$ = -8.13, 95% CI [-9.33, -6.94] ;
$\beta_{[\text{Maternal age 2}]}$ = -5.94, 95% CI [-7.20, -4.67];
$\beta_{[\text{Maternal age 3}]}$ = -8.79, 95% CI [-10.45, -7.14];
$\beta_{[\text{Maternal age 4}]}$ = -4.82, 95% CI [-7.52, -2.11];
$\beta_{[\text{Maternal age 5}]}$ = -5.04, 95% CI [-7.93, -2.14].

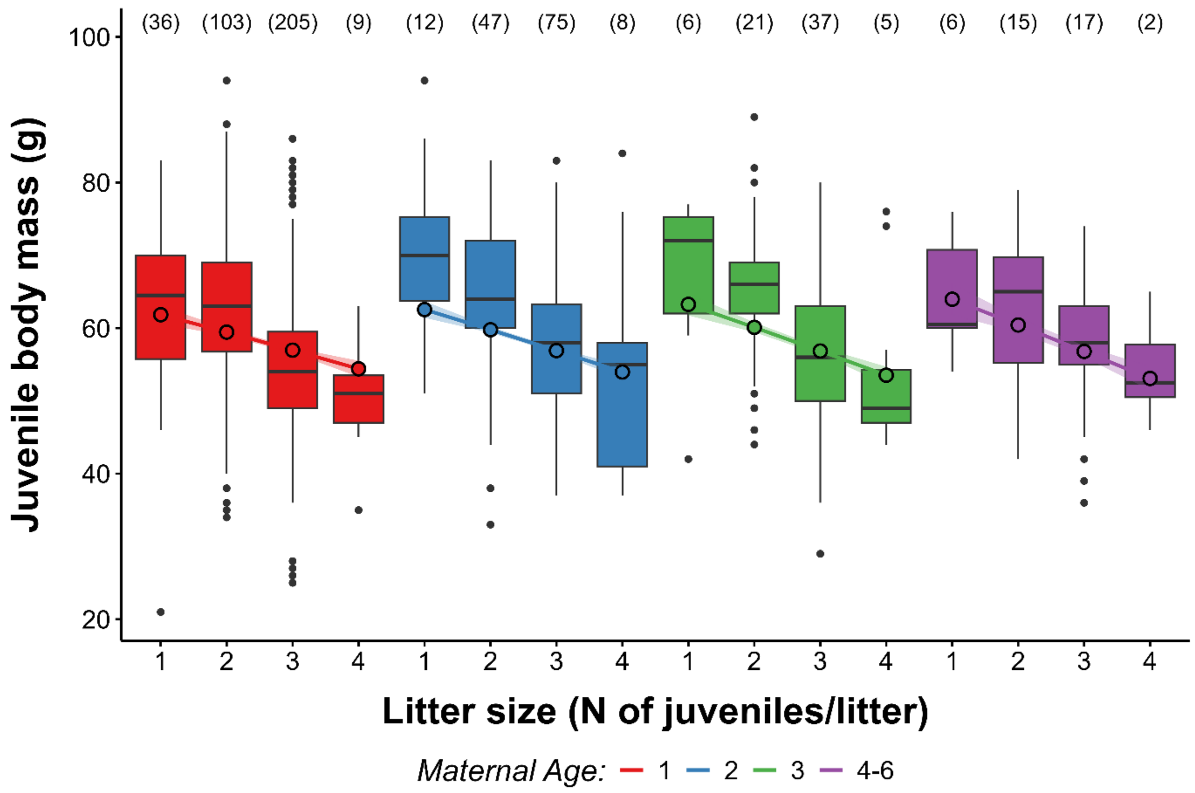

Figure S6. Variation in juvenile body mass–litter size trade-off according to maternal age (in years). Females of ages 4-6 were grouped together in this figure. Lines and shades correspond to the prediction of the top model ‘Age * N_{juv} + N_{juv}^2’ (Table S6) and the 95% confidence intervals, respectively. The number of unique females for each combination of maternal age and litter size is shown at the top.

6) Maternal survival with Capture-Mark-Recapture models

Goodness of fit - We carried out several GOF-tests to test the fit of data to a Cormack-Jolly-Seber model using the R2ucare package (Gimenez et al., 2018). We tested the assumptions of equal survival (Test3.SR, Test3.SM) and equal catchability of marked individuals (Test2.CT and Test2.CL) at each study site, Luoto and Vaasa. We found no compelling evidence for trap-dependence or transience effects on survival, as neither the overall GOF-test nor any of TEST 2 and 3 were significant. The variance inflation factor (c-hat) was estimated as the ratio of the overall $\chi 2$ statistic and the degrees of freedom. No overdispersion was detected but rather underdispersion (c-hat = 0.90 and 0.65 without or with site differences, respectively). We applied a conservative approach for variance adjustment (i.e., c-hat = 1; Cooch & White, 2014). In addition, we performed transience and trap-dependence tests for females marked as juveniles and females marked as adults, and found no violation of these assumptions.

Table S7. Goodness of fit tests for trap-dependence and transience effect at each site (Vaasa and Luoto). With χ^2, the statistic of the test and df, the degrees of freedom.

		**Luoto**			**Vaasa**			**All sites**		
	**Test**	χ^2	**df**	**p-value**	χ^2	**df**	**p-value**	χ^2	**df**	**p-value**
**Transience tests**	**3SR**	21.76	20	0.35	14.45	21	0.85	27.77	21	0.15
	**3SM**	0.94	7	0.996	0	8	1	2.05	11	0.998
**Trap-dependence tests**	**2CT**	3	1	0	5.04	6	0.54	6.36	8	0.61
	**2CL**	0	0	1	0	0	1	0	0	1

Model selection - We analysed 570 female capture history data collected from 1992 to 2014 with Cormack-Jolly-Seber models in RMark (Laake, 2013). Starting from the general model *phi*(time)*p*(time), we changed the structure for p by testing age and group effects (site, time period, age at marking, birth and annual food availabilities) on recapture probabilities (*p*), then used the most-parsimonious models of *p* to model the survival probability (*phi;* Table S8). Model selection was based on Akaike's information criterion adjusted for small sample size (AICc). If competing models were found (ΔAICc < 2), the most parsimonious model was chosen. We used age as a continuous variable and constrained the trajectory of survival at each age as a Gompertz or logit-linear functions. Then, a threshold modelling and model selection were applied to estimate the onset of senescence (at age 1 to 5, five scenarios, Table S9), using Gompertz age trajectory of female adult survival.

Table S8. Results of the selection procedure on recapture (*p*) parameters using age as a categorical variable or continuous variable (logit and loglog link). Annual variation in survival is considered: *phi***(~time)**. The number of parameters (np), the Akaike's Information Criterion corrected for small sample sizes (AICc), ΔAICc, model weight (ω) and deviance are presented.

NAME	np	AICc	ΔAICc	ω	Deviance
**Selection for *p***					
***Phi*(~time) p(~effort)**	**24**	**1364.67**	**0.00**	**0.38**	**424.52**
***Phi*(~time) p(~site + effort)**	**25**	**1366.01**	**1.33**	**0.20**	**423.74**
***Phi*(~time) p(~1)**	**23**	**1366.51**	**1.84**	**0.15**	**428.47**
Phi(~time) *p*(~MarkingAge)	24	1367.98	3.31	0.07	427.83
Phi(~time) *p*(~BirthP)	24	1368.56	3.88	0.06	428.40
Phi(~time) *p*(~site)	24	1368.61	3.93	0.05	428.45
Phi(~time) *p*(~AdP)	24	1368.62	3.95	0.05	428.47
Phi(~time) *p*(~age_cat + effort)	29	1372.06	7.39	0.01	421.28
Phi(~time) *p*(~site + effort + age_cat)	30	1373.05	8.37	0.01	420.13
Phi(~time) *p*(~age_cat)	28	1373.09	8.42	0.01	424.45
Phi(~time) *p*(~site + effort + age_cat + BirthP)	31	1374.76	10.09	0.00	419.70
Phi(~time) *p*(~site + age_cat)	29	1374.94	10.27	0.00	424.16
Phi(~time) *p*(~site + effort + age_cat + AdP)	31	1375.19	10.51	0.00	420.13
Phi(~time) *p*(~site + effort + age_cat + BirthP + AdP)	32	1376.90	12.23	0.00	419.69
Phi(~time) *p*(~time)	44	1385.95	21.28	0.00	402.56

Abbreviations: effort = capture efforts during two monitoring periods (1992-2000 vs 2001-2014); BirthFood = food availability at birth (individual covariate); AnnualFood = food availability at year t; site = site effect (Vaasa and Luoto); MarkingAge = marked as juveniles or as adults (yearlings); time = years of monitoring.

Table S9. AICc model selection for age-specific variation in survival (*phi*). Age at marking, site and annual food availability at adulthood were included as covariates. Threshold_AgeX_a = threshold model with a breakpoint at age X, with $\beta_{Age.pre-onset}=0$ and $\beta_{Age.post-onset}$) and Threshold_AgeX_b = threshold model with a breakpoint at age X, with slopes $\beta_{Age.pre-onset}$ and $\beta_{Age.post-onset} \neq 0$). The number of parameters (np), the Akaike's Information Criterion corrected for small sample sizes (AICc), ΔAICc, model weight (ω) and deviance are presented.

NAME		np	AICc	ΔAICc	ω	Deviance
a) Selection on age for *phi*						
***Phi*(~Threshold_Age3_a)**	***p*(~1)**	**6**	**1380.34**	**0.00**	**0.29**	**477.45**
***Phi*(~Threshold_Age2_a)**	***p*(~1)**	**6**	**1381.20**	**0.86**	**0.19**	**478.31**
Phi(~Threshold_Age3_b)	*p*(~1)	7	1382.36	2.02	0.10	477.44
Phi(~Age + Age^2)	*p*(~1)	7	1382.68	2.35	0.09	477.77
Phi(~Threshold_Age2_b)	*p*(~1)	7	1382.88	2.55	0.08	477.96
Phi(~Age)	*p*(~1)	6	1383.20	2.86	0.07	480.31
Phi(~Threshold_Age4_a)	*p*(~1)	6	1383.50	3.17	0.06	480.62
Phi(~Threshold_Age4_b)	*p*(~1)	7	1384.22	3.88	0.04	479.30
Phi(~Threshold_Age5_b)	*p*(~1)	7	1384.73	4.40	0.03	479.81
Phi(~1)	*p*(~1)	5	1385.22	4.89	0.03	484.36
Phi(~Threshold_Age5_a)	*p*(~1)	6	1385.38	5.04	0.02	482.49
b) Effect of food availability experienced by the mothers at birth						
Phi(~Threshold_Age3_a + BirthFood)	*p*(~1)	**7**	1379.79	0.00	0.42	1365.67
Phi(~Threshold_Age3_a)	*p*(~1)	6	1380.34	0.54	0.32	477.45
Phi(~Threshold_Age3_a * BirthFood)	*p*(~1)	8	1380.79	0.99	0.26	1364.63

Table S10. AICc model selection for *phi* using 'time' (years of monitoring from 1992 to 2014) as a covariate. The number of parameters (np), the Akaike's Information Criterion corrected for small sample sizes (AICc), ΔAICc, model weight (ω) and deviance are presented.

Models on Phi	***p***	**np**	**AICc**	**ΔAICc**	**ω**	**Dev.**
**time + Threshold_Age3_a + AnnualFood + MarkingAge + site**	**p(~1)**	**27**	**1363.90**	**0.00**	**0.23**	**417.38**
**time + Threshold_Age2_a + AnnualFood + MarkingAge + site**	**p(~1)**	**27**	**1364.95**	**1.06**	**0.14**	**418.44**
**time + AnnualFood + MarkingAge** + site	**p(~1)**	**25**	**1365.03**	**1.13**	**0.13**	**422.76**
time + Threshold_Age3_b + AnnualFood + MarkingAge	p(~1)	28	1366.02	2.12	0.08	417.37
time	p(~1)	23	1366.51	2.62	0.06	428.47
time + Age + AnnualFood + MarkingAge + site	p(~1)	27	1366.53	2.63	0.06	420.02
time + Age + Age^2 + AnnualFood + MarkingAge + site	p(~1)	28	1366.69	2.80	0.06	418.05
time + Threshold_Age2_b + AnnualFood + MarkingAge + site	p(~1)	28	1366.70	2.80	0.06	418.05
time + Threshold_Age4_a + AnnualFood + MarkingAge + site	p(~1)	27	1366.92	3.02	0.05	420.40
time + AnnualFood + MarkingAge + site	p(~1)	26	1367.13	3.23	0.05	422.74
time + Threshold_Age4_b + AnnualFood + MarkingAge + site	p(~1)	28	1368.10	4.20	0.03	419.45
time + Threshold_Age5_a + AnnualFood + MarkingAge + site	p(~1)	27	1368.49	4.60	0.02	421.98
time + Threshold_Age5_b + AnnualFood + MarkingAge + site	p(~1)	28	1368.58	4.68	0.02	419.93
1	p(~1)	2	1385.35	21.45	0.00	490.54

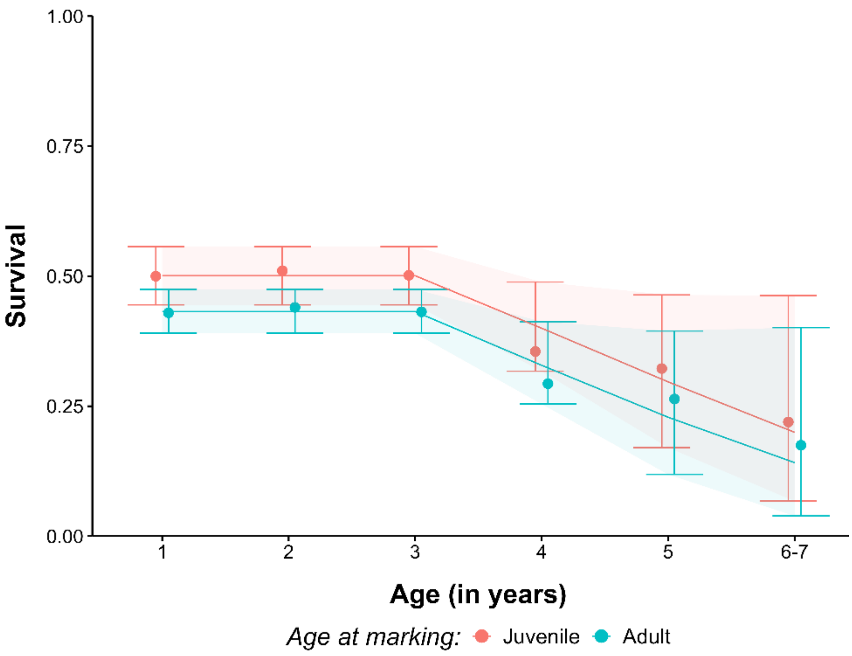

Figure S7. Age-dependent estimates of annual female survival probabilities (circles; 95% confidence intervals are indicated by vertical bars) according to age at marking (marked as juvenile or adult in red and blue, respectively; estimates from model *phi*(age + MarkingAge)*p*(.) with age a categorical variable). Lines represent the estimates from model *Phi*(~Threshold_Age3 + MarkingAge) *p*(~1), assuming a Gompertz function and an onset of senescence at three years old (shades indicate 95% confidence intervals).

References

Cooch, E. G., & White, G. C. (2014). *Program MARK - A Gentle Introduction* (13th ed.). http://www.phidot.org/software/mark/docs/book

Gallego-Zamorano, J., Hokkanen, T., & Lehikoinen, A. (2016). Climate-driven synchrony in seed production of masting deciduous and conifer tree species. *Journal of Plant Ecology*, *11*(2), 180–188. https://doi.org/10.1093/jpe/rtw117

Gimenez, O., Lebreton, J., Choquet, R., & Pradel, R. (2018). R2ucare: An <scp>r</scp> package to perform goodness-of-fit tests for capture–recapture models. *Methods in Ecology and Evolution*, *9*(7), 1749–1754. https://doi.org/10.1111/2041-210X.13014

Hokkanen, T. (2000). Seed crops and seed crop forecasts for a number of tree species. In *Forest regeneration in the northern parts of Europe* (pp. 87–97). The Finnish Forest Research Institute: Papers, 790.

Laake, J. L. (2013). *RMark: An R Interface for Analysis of Capture-Recapture Data with MARK.*

Ranta, H., Hokkanen, T., Linkosalo, T., Laukkanen, L., Bondestam, K., & Oksanen, A. (2008). Male flowering of birch: Spatial synchronization, year-to-year variation and relation of catkin numbers and airborne pollen counts. *Forest Ecology and Management*, *255*(3–4), 643–650. https://doi.org/10.1016/j.foreco.2007.09.040
